# Glutamate, Ornithine, Arginine, Proline, and Polyamine Metabolic Interactions: The Pathway Is Regulated at the Post-Transcriptional Level

**DOI:** 10.3389/fpls.2016.00078

**Published:** 2016-02-16

**Authors:** Rajtilak Majumdar, Boubker Barchi, Swathi A. Turlapati, Maegan Gagne, Rakesh Minocha, Stephanie Long, Subhash C. Minocha

**Affiliations:** ^1^Department of Biological Sciences, University of New HampshireDurham, NH, USA; ^2^United States Department of Agriculture Forest Service, Northern Research StationDurham, NH, USA

**Keywords:** *Arabidopsis thaliana*, arginine, γ-aminobutyric acid, glutamate, ornithine, polyamines, proline, quantitative polymerase chain reaction

## Abstract

The metabolism of glutamate into ornithine, arginine, proline, and polyamines is a major network of nitrogen-metabolizing pathways in plants, which also produces intermediates like nitric oxide, and γ-aminobutyric acid (GABA) that play critical roles in plant development and stress. While the accumulations of intermediates and the products of this network depend primarily on nitrogen assimilation, the overall regulation of the interacting sub-pathways is not well understood. We tested the hypothesis that diversion of ornithine into polyamine biosynthesis (by transgenic approach) not only plays a role in regulating its own biosynthesis from glutamate but also affects arginine and proline biosynthesis. Using two high putrescine producing lines of *Arabidopsis thaliana* (containing a transgenic mouse *ornithine decarboxylase* gene), we studied the: (1) effects of exogenous supply of carbon and nitrogen on polyamines and pools of soluble amino acids; and, (2) expression of genes encoding key enzymes in the interactive pathways of arginine, proline and GABA biosynthesis as well as the catabolism of polyamines. Our findings suggest that: (1) the overall conversion of glutamate to arginine and polyamines is enhanced by increased utilization of ornithine for polyamine biosynthesis by the transgene product; (2) proline and arginine biosynthesis are regulated independently of polyamines and GABA biosynthesis; (3) the expression of most genes (28 that were studied) that encode enzymes of the interacting sub-pathways of arginine and GABA biosynthesis does not change even though overall biosynthesis of Orn from glutamate is increased several fold; and (4) increased polyamine biosynthesis results in increased assimilation of both nitrogen and carbon by the cells.

## Introduction

**Figure 1:**
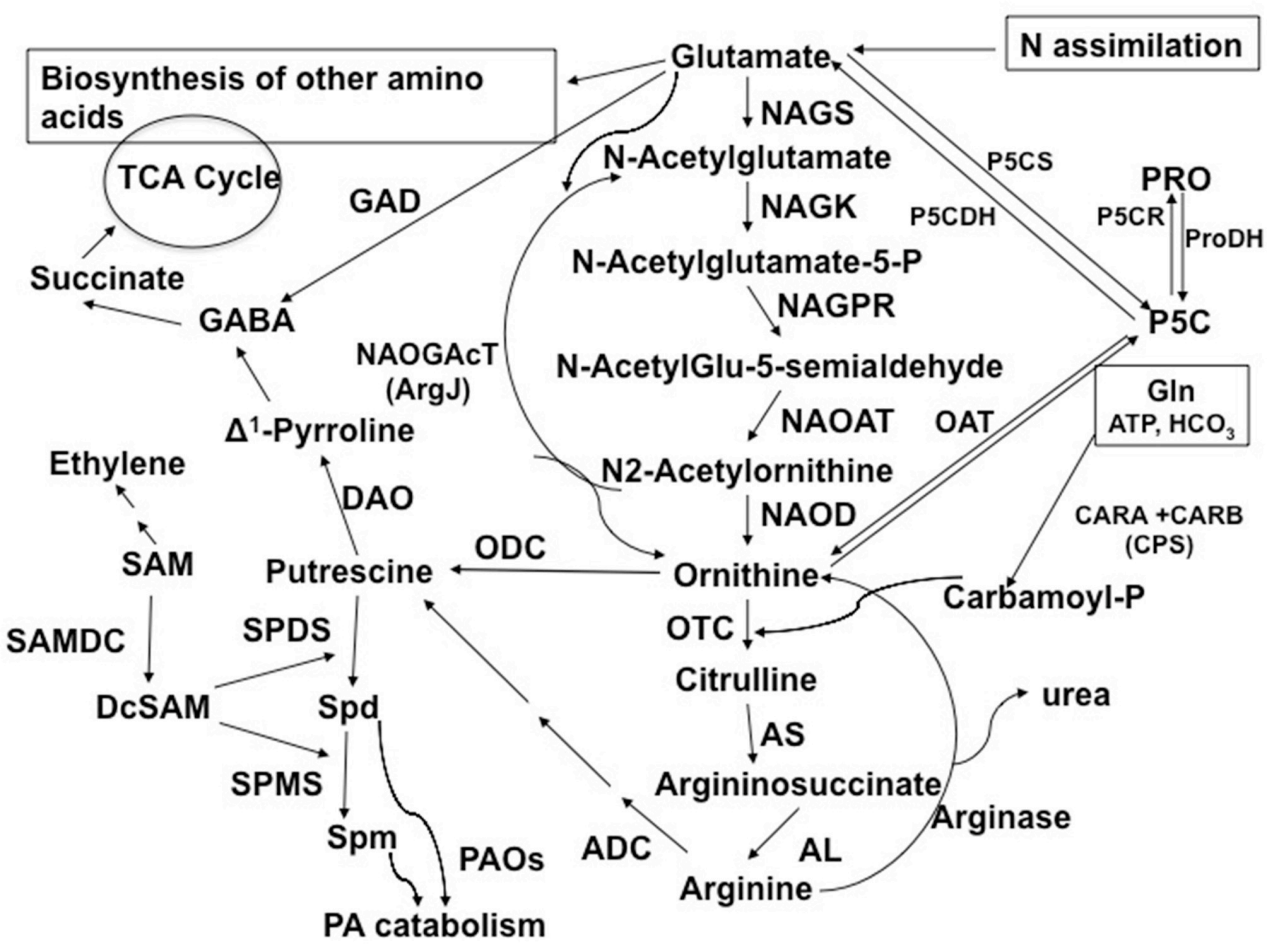
**Abbreviated pathway for the biosynthesis and metabolism of Orn in plants in connection to polyamines, amino acids, TCA cycle metabolites, and alkaloids**. Abbreviations and EC numbers of the enzymes: ADC, arginine decarboxylase (EC 4.1.1.19); AL, argininosuccinate lyase (EC 4.3.2.1); AS, argininosuccinate synthase (EC 6.3.4.5); CPS (CARA and CARB, carbamoylphosphate synthetase (EC 6.3.5.5); DAO, diamine oxidase (EC 1.4.3.22); GAD, glutamate decarboxylase (EC 4.1.1.15); NAGK, *N*-acetylglutamate kinase (EC 2.7.2.8); NAGPR, *N*-acetylglutamate-5-phosphate reductase (EC 1.2.1.38); NAGS, *N*-acetylglutamate synthase (EC 2.3.1.1); NAOAT, N-acetylornithine aminotransferase (EC 2.6.1.11); NAOD, N-acetylornithine deacetylase (EC 3.5.1.16); NAOGAcT, *N*_2_-Acetylornithine:Glu-acetyl transferase or ArgJ (EC 2.3.1.35); OAT, ornithine δ-aminotransferase (EC 2.6.1.13); ODC, ornithine decarboxylase (EC 4.1.1.17); OTC, ornithine transcarbamoylase (EC 2.1.3.3); ProDH, proline dehydrogenase (EC 1.5.99.8); P5CDH, Δ^1^-pyrroline-5-carboxylate dehydrogenase (EC 1.5.1.12); P5CR, Δ^1^-pyrroline-5-carboxylate reductase (EC 1.5.1.2); P5CS, Δ^1^-pyrroline-5-carboxylate synthetase (EC 2.7.2.11/1.2.1.41); SAMDC, *S*-adenosylmethionine decarboxylase (EC 4.1.1.50); SPDS, spermidine synthase (EC 2.5.1.16); PAO, polyamine oxidase (EC 1.5.3.11); SPMS, spermine synthase (EC 2.5.1.22). Modified from Majumdar et al. (2015).

The glutamate (Glu) to proline (Pro), ornithine (Orn), arginine (Arg), polyamines (PAs), and γ-aminobutyric acid (GABA) group of reactions constitutes one of the major interactive pathways for carbon (C) and nitrogen (N) assimilation and partitioning (Figure 1). The products of this pathway have a wide range of physiological functions in plants. In addition to the production of amino acids and signal molecules like GABA and nitric oxide (NO), this group of sub-pathways is the primary source of putrescine (Put) biosynthesis, which in turn produces the other two common PAs, spermidine (Spd) and spermine (Spm). The three PAs are obligatory requirements for cell survival and growth through their molecular interactions with nucleic acids (transcription and translation) and cellular membranes (Kusano et al., 2007; Minocha et al., 2014); they are also a source of GABA, which plays critical roles in diverse cellular functions in plants (Shelp et al., 2012a). Interactions of PAs with polyanionic macromolecules and cellular membranes (Wallace et al., 2003), and with hydroxycinnamic acids, fatty acids or alkaloids underlie some of their roles in abiotic and biotic stress responses (Flores and Filner, 1985; Martin-Tanguy, 1997; Ghosh, 2000; Bagni and Tassoni, 2001; Subramanyam et al., 2015).

Cellular accumulation of PAs is the net result of their biosynthesis, inter-conversions, catabolism and conjugation (the last one particularly in mature tissues and organs); their biosynthesis is controlled by enzyme activity as well as the availability of substrates. The two pathways of Put biosynthesis (Figure 1) appear to be independently regulated by separate enzymes, namely Arg decarboxylase (ADC – EC: 4.1.1.19) and Orn decarboxylase (ODC – EC: 4.1.1.17). Spermidine and Spm biosynthesis is controlled by a combination of S-adenosylmethionine decarboxylase (SAMDC, *a.k.a.* AdoMet DC – EC: 4.1.1.50) and two aminopropyltransferases, namely Spd synthase (SPDS – EC 2.5.1.16) and Spm synthase (SPMS – EC 2.5.1.22; reviewed in Shao et al., 2014). Additionally, it is known that the diversion of SAM toward PAs (e.g., via transgenic expression of yeast *SPDS* and *SAMDC* genes in tomato fruits) can enhance the metabolic interactions (cf. competition) of PAs and ethylene (C_2_H_4_) biosynthesis, and delay fruit ripening and senescence, thereby increasing the shelf life of the fruit (Nambeesan et al., 2010; Lasanajak et al., 2014). The catabolism of PAs, which produces GABA (a metabolite of great significance for its positive role in the oxidative stress response—Shi et al., 2010; Vergara et al., 2012), and H_2_O_2_ in the apoplast (for cell wall lignin biosynthesis), is also involved in maintaining the balance of C:N in plants (Bouché and Fromm, 2004; Fait et al., 2008). Further complexity of cellular PA functions involves their interactions with plasma membrane cellular H^+^ pumps (Garufi et al., 2007) and the transport of Ca^2+^ and K^+^ across root membranes in a species-specific manner (Zepeda-Jazo et al., 2011).

Due to their pleiotropic functions, regulation of PA homeostasis is complex (Agostinelli, 2014). Several recent studies have shown that homeostatic up-regulation of Put biosynthesis (e.g., via transgenic approaches) leads to widespread metabolic consequences affecting several amino acids, sugars, sugar alcohols, phytochelatins, organic acids and inorganic ions (Minocha et al., 2004; Mattoo et al., 2010; Mohapatra et al., 2010a,b; Page et al., 2012; Majumdar et al., 2013). Arginine, Pro, GABA and Put concentrations in plants are among the known indicators of various forms of abiotic stress in herbaceous annuals, as well as woody perennials (Ericsson et al., 1993, 1995; Näsholm et al., 1994, 1997, 2000; Wargo et al., 2002; Mohapatra et al., 2010b; Minocha et al., 2013, 2015).

Glutamate → Orn → Arg, Glu → Pro, and Orn → Pro are largely reversible linear pathways, while Put production is a branched irreversible pathway using Orn and Arg as substrates; this pathway also leads to the production of Spd, Spm, and GABA (Figure 1). In addition, GABA is synthesized directly from Glu by the enzyme Glu decarboxylase (GAD – EC: 4.1.1.15). Although there is abundant literature on GABA biosynthesis and its physiological functions, specific contributions of the direct (Glu → GABA) *vis-a-vis* indirect (Glu → Orn/Arg → Put → GABA) pathways of its biosynthesis are not known (Shelp et al., 2012a; Trobacher et al., 2013; Hu et al., 2015). Likewise, regulation of the flux of Glu into Orn/Arg/Put and Pro under conditions of increased need for the biosynthesis of Put (e.g., due to abiotic stress response or experimental up-regulation of Put production via transgenic approaches) is still enigmatic. Equally puzzling is the mechanism by which the multi-step process of Glu → Orn/Arg is regulated. Our previous studies with genetically engineered poplar (*Populus nigra x maximowiczii*—clone NM6) cell cultures for high Put production [via transgenic expression of a mouse *ODC* (m*ODC*), which produces Put from Orn] have suggested that the need for increased Orn production may be met without induction (i.e., transcription) of most of the enzymes of the Glu → Orn pathway (Page et al., 2007, 2012).

The research presented here was aimed at investigating the effects of perturbation of the Glu → Orn → Arg → Put, Glu → Orn → Pro, Arg → Orn → Glu and Pro pathway into PAs by creating a new path (a short cut) for redirecting Orn toward Put, something that *A. thaliana* does not naturally do. The transgenic production of a m*ODC* enzyme, which has a rather low Km (<100 μM) for Orn (Coleman et al., 1993), efficiently converts large amounts of Orn into Put (Descenzo and Minocha, 1993; Bastola and Minocha, 1995; Bhatnagar et al., 2001; Majumdar et al., 2013), which can be stored in plants in relatively large (mM) concentrations.

We report here the results of our study involving: (1) the effects of supplementary N and C application on cellular PAs and amino acids in the wild type (WT) and transgenic plants of *Arabidopsis thaliana*, which produce several-fold higher concentrations of Put via the constitutive (2x*35S* CaMV promoter) expression of m*ODC* gene (cDNA); and (2) the effects of inducible expression of m*ODC* (transient increase in Put production) on the expression of genes encoding several enzymes of the Orn → Arg, Orn⇔Pro, Glu/PAs → GABA pathways, as well as those involved with initial steps in the PA catabolism pathway. The aim was to assess if one or more specific step (or steps) in the pathway is (are) transcriptionally triggered in response to increased consumption of Orn (or increased production of Put) via constitutive or inducible production of m*ODC*. We also report on the accumulation of biomass and changes in chlorophyll and total N and C per plant in response to additional supply of N and C to the plants.

## Materials and methods

### Transgenic *Arabidopsis thaliana* plants

The m*ODC* cDNA that was earlier used for poplar cell transformation (Bhatnagar et al., 2001) was PCR amplified and cloned into pCR8.0/GW/TOPO or pENTR™/D-TOPO® vector (Invitrogen, Carlsbad, CA), and subsequently transferred into Gateway-compatible pMDC32 vector (containing 2x*35S* promoter) and pMDC7 vector (containing estradiol inducible promoter—Curtis and Grossniklaus, 2003; Majumdar et al., 2013), respectively. The resultant vectors with the m*ODC* coding sequence were used to transform *A. thaliana* (Columbia-0) plants to obtain constitutive and inducible m*ODC* transgenic lines (Majumdar et al., 2013). These lines were characterized for the presence of the transgenes (hygromycin resistance—*HPTII* for selection of transformants and the m*ODC* gene) by Polymerase Chain Reaction (PCR), their transcription (by Reverse Transcriptase PCR), and for their PA contents by High Performance Liquid Chromatography (HPLC). Third or fourth generation (T_3_ or T_4_ homozygous for the m*ODC* gene) seeds homozygous for the two transgenes were used for all experiments.

### Growth conditions and treatments

Arabidopsis seeds (T_3_) were surface sterilized with ethanol, dried under the laminar flow hood, and grown on Petri dishes with solid germination medium (GM) containing 4.3 g.L^−1^ of Murashige and Skoog (1962) premix salt powder (MS) plus Gamborg's organics (Gamborg et al., 1968), 0.5 g.L^−1^ of 2-(N-Morpholino) ethanesulfonic acid (MES), 1% (~30 mM) sucrose, and 0.8% type A agar (Sigma Aldrich, St. Louis, MO), and adjusted to pH 5.7. For N and sucrose treatments, seeds were germinated on: (i) GM supplemented with either 30 mM or 60 mM additional KNO_3_ (regular GM contains 39.43 mM NO_3_), (ii) modified GM without N (i.e., MS without NH_4_NO_3_ and KNO_3_ but with additional KCl to maintain equimolar concentration of K), and (iii) GM without sucrose or containing 20 or 70 mM additional concentration of sucrose. Following cold (4°C) treatment (in the dark) for 2 days, the seeds in Petri dishes were transferred to a walk-in growth chamber at 25 ± 1°C under 12/12 h photoperiod (80 ± 10 μE m^−2^.s^−1^). At 12 days, batches of seedlings (7–8 per batch) were collected for fresh weight (FW) and dry weight (DW) analysis in glass vials, and for PA and amino acids analyses in 5% perchloric acid (PCA, ~ 0.77N – 9 μL.mg^−1^ FW). The former were dried at constant temperature (70°C) in a drying oven and the latter were stored at −20°C prior to analysis of PAs and amino acids (AAs).

For short-term m*ODC* induction experiments, 2 week-old seedlings (from T_4_ seeds) of an inducible m*ODC* line germinated on solid GM were transferred to 12 well (4 columns × 3 rows) culture plates. Each well contained 15–20 seedlings (3 replicate wells for each treatment) resting in 1 mL of liquid GM plus 5 μM estradiol (inducer; Sigma-Aldrich) or no estradiol (control treatment). Replicate wells of the same treatment were arranged in a column and induced vs. un-induced plants of the same genotype were kept in adjacent wells. Thus, the WT and transgenic plants were kept in different 12-well culture plates. All plates were kept in the walk-in growth chamber under conditions described above. Seedlings were collected at 24 and 48 h after induction/treatment. At the time of collection, seedlings were placed on paper towels for a few seconds to remove excess liquid, and ~50 mg FW (8–10 whole seedlings) samples were collected in triplicate in microfuge tubes and mixed with 5% PCA (9 μL 5% PCA per mg FW seedlings), and stored at −20°C for PA analyses.

### Plant growth in soil

Arabidopsis seeds (T_4_ generation) were sown in moist soil mix containing 3 parts Scott's 360 Metro-Mix (Scotts Company, Marysville, OH) and 1 part perlite in 3″ pots. In each pot, 10–15 seeds were planted and the pots were placed in a plastic tray covered with a clear plastic lid; the trays were kept for 48 h in the dark at 4°C. Thereafter, the trays were moved to a walk-in growth chamber at 21°C under 18 h photoperiod (80 ± 10 μE m^−2^s^−1^). Plants were watered on alternate days and supplied with the addition of ¼strength Miracle-Gro (Scotts Company) synthetic fertilizer in the irrigation water every 5th day. Two weeks after germination, the plants were thinned to two plants per pot.

The foliage of 5 week-old WT and m*ODC*-1-7 transgenic (constitutive) plants grown in pots (3 pots/treatment for both WT and m*ODC* plants; each pot containing 2 plants) were sprayed with two different N fertilizers (each at 2% concentration): urea (20-0-0) or Nitamin® (30-0-0; http://www.kochagronomicservices.com/downloads/d682.aspx?type=view) or pure water, all containing 0.05% Silwet surfactant (http://www.helenachemical.com/products/utility/silwet-l77/). For PA analysis, leaves (3–4 leaves to yield ~100 mg FW) were collected in 5% PCA (3 replicates) at 1, 2, 5, and 8 days after treatment. For DW analysis, whole plants were collected (three replicates) from each treatment after 8 days, weighed, oven dried (at 80°C for 48 h) and weighed again.

### Quantification of soluble protein, chlorophyll and total C and N

Chlorophyll analysis was performed using a modified protocol from Gitelson et al. (2009). Two to three healthy rosette leaves from outer whorls of 4 week-old-plants were weighed and homogenized in MeOH (200 μL mg^−1^ FW) using a mortar and pestle. Ten to twenty mg of CaCO_3_ was added to the samples while grinding to prevent pheophytization of chlorophyll. Homogenates were centrifuged for 5 min at 10,000 xg and the A_665_ and A_652_ of the supernatants were measured (Spectronic Instruments Inc., Rochester, NY). Chlorophyll a and chlorophyll b concentrations were calculated as per Lichtenthaler and Buschmann (1987).

For the analyses of total C and N, individual plants were collected separately, dried at 70°C, and analyzed using a CE Elantech Flash EA1112 combustion NC Soil analyzer (Thermo Scientific, Lakewood, NJ) according to EPA method 440.0 using NIST (National Institute of Standards and Technology, Gaithersburg, MD, USA) standard reference materials 1515 (apple leaves) and 1547 (peach leaves) for procedure verification (Mohapatra et al., 2010a).

Total soluble protein concentration was analyzed in tissue extracts in potassium phosphate buffer (0.1 M; pH 7.0) by the Bradford (1976) method using bovine serum albumin as standard.

### Quantification of polyamines and amino acids

Polyamines and amino acids were analyzed in samples stored in 5% PCA by dansylation and HPLC. Plant samples in PCA were subjected to three cycles of freezing at −20°C and thawing at room temperature before dansylation. The extracts, after the final thawing, were vortexed for 1 min and centrifuged for 5 min at 14,000 xg. One hundered μL of the supernatant from each sample and 5 standards (mixture of 3 PAs) with 20 μL of 0.1 mM heptanediamine as the internal standard were dansylated as per Minocha and Long (2004) using 50 μL of 20 mg mL^−1^ Asn (in water) to remove the unreacted dansyl chloride. The dansyl-PAs were extracted in 400 μL of toluene by partitioning. Aliquots of 200 μL from the toluene fraction were transferred into new microfuge tubes and vacuum dried. Dansyl-PAs were dissolved in 500 μL of methanol and transferred into autosampler vials for analysis by HPLC. The HPLC system included a series 200 autosampler (PerkinElmer Inc., Waltham, MA), quaternary pump and fluorescence detector fitted with a Pecosphere C_18_ reversed phase cartridge column (4.6 × 33 mm, 3 μm). The detector was set at excitation and emission wavelengths of 340 and 515 nm, respectively. Ten or 20 μL of standards and samples were injected and separated using a 40% acetonitrile (in10 mM heptane sulfonic acid) to 100% acetonitrile in a linear gradient at a flow rate of 2.5 mL min^−1^. The data were integrated using Perkin Elmer TotalChrom software (version 6.2.1). Amino acids were analyzed by HPLC as per Minocha and Long (2004).

### Statistical analysis of biochemical data

For all experiments, typically three biological replicates were used per treatment. Each experiment was repeated at least twice and data from a single representative experiment are presented here. The data were analyzed using one-way ANOVA and Tukey's test for comparison between the treatment and the respective control as indicated specifically in Figure legends. Analyses were done using SYSTAT Version 10.2 for Windows (Systat Software, Inc., San Jose, CA 95131 USA) and Microsoft Excel (Version 2010); significant difference between treatment and control were analyzed at *P* ≤ 0.05.

### Analysis of gene expression by qPCR—preparation of RNA and cDNA

Two separate experiments were conducted for analysis of gene expression, which involved slightly different procedures. In one experiment, 28 genes were tested using a single set of homozygous plants (T_3_ generation) from which RNA was isolated once and cDNA was prepared twice. The procedures for RNA isolation, cDNA preparation and qPCR analysis data for this experiment are presented as Supplemental Material. The second experiment involved the following procedure, but was used only for 15 genes (including m*ODC*); the same transgenic line was used for this experiment.

At 14 days post germination, batches of 12–15 seedlings were transferred from the germination plates into 12-well plates. Each batch of seedlings placed in a well was considered one replicate and each 12-well plate contained all treatments. Treatment position was randomly allocated within each block following a randomized complete block design with 4 replicates. Each well contained 1 mL of liquid GM. The seedlings were allowed to acclimate overnight in the growth chamber. Induction of m*ODC* was accomplished by adding a final concentration of 5 μM estradiol (from 10 mM stock dissolved in dimethyl sulfoxide). Samples were collected at 24 and 48 h after induction for PA quantification (by HPLC) as well as for RNA extraction (frozen in liquid nitrogen and at −80°C).

RNA extractions were performed maintaining the block structure of the experiment. Frozen tissue was homogenized in liquid nitrogen using disposable RNase-free pestles and a cordless pestle motor (Thermo-Fisher Scientific, Waltham, MA). While tissue was still frozen, Tri reagent (Thermo-Fisher) was added at a ratio of 1 mL to 100 mg of tissue. Samples were incubated at room temperature for 5 min, then centrifuged at 12,000 xg for 10 min at 4°C. The clear homogenate was transferred to a new tube and chloroform was added at a ratio of 0.2 mL for 1 mL of Tri reagent originally added. Tubes were shaken aggressively, incubated at room temperature for 3 min, and centrifuged at 12,000 xg for 15 min at 4°C. The upper aqueous phase was removed and mixed with half a volume of isopropanol. Samples were inverted 10 times and frozen at −20°C overnight. RNA was pelleted by centrifuging at 12,000 xg for 10 min at 4°C. The pellets was washed with 75% ethanol by centrifugation, dried for 10 min at room temperature, re-suspended in RNase-free water, and analyzed on the Agilent 2200 TapeStation with the RNA ScreenTape kit (Agilent Technologies, Santa Clara, CA). Three high quality samples with clear 18S, 28S, 16S, and 23S peaks were used for cDNA preparation.

First strand cDNA synthesis was done using High-Capacity RNA to cDNA kit (Applied Biosystems, Grand Island, NY) following the manufacturer's protocol. The final reaction volume of 20 μL contained 10 μL of 2X RT Master Mix and 10 μL of RNA sample (up to 2 μg of total RNA). The reaction was set up in an Eppendorf Mastercycler® Nexus PCR thermal cycler (Eppendorf NA, Hauppauge, NY) with the following conditions: 60 min at 37°C and 5 min at 95°C, hold at 4°C. The cDNA was stored at −20°C for later use in qPCR analysis. All gene expression analyses reported here were done from the same RNA preparation and one set of cDNA preparation.

### Quantitative polymerase chain reaction (qPCR)

RT-PCRs were conducted by using 1 μl of cDNA as initial template, 200 nM concentration of each gene specific forward and reverse primer combination (Supplemental Table 1), and One-Taq® Quick-Load® 2X Mastermix with standard Buffer (New England Biolabs, Ipswich, MA) in Eppendorf Mastercycler (Eppendorf Corp, Enfield, CT) following standard cycling conditions (denaturation at 94°C for 30 s followed by 35 cycles of denaturation at 94°C for 30 s, annealing for 30 s at 58°C and extension step at 68°C for 1 min). The amplified products were electrophoresed on 1% agarose gels and the size of the amplicons was verified with the published literature.

The qPCR standards were made as follows: mix 2 μL aliquot from each cDNA sample, and make seven 4-fold serial dilutions. Primer efficiencies and dissociation curve analyses were conducted by using Fast SYBR® Green Master Mix (Applied Biosystems, Grand Island, NY) following the manufacturer's protocol. Briefly, 5.5 μL of Fast SYBR green, and a final 300 nM concentration of each of the gene-specific forward and reverse primers (Supplemental Table 1), and 4 μL volume from each standard were mixed in a final reaction volume of 10 μL. The reactions were run with two technical replicates per biological replicate in non-skirted natural 96-well reaction plates (MIDSCI, Valley Park, MO), using Quantitative PCR, SYBR green dissociation curve method (Stratagene MxPro 3000 qPCR Thermocycler). The qPCR reactions were conducted with the following conditions: initial denaturation at 95°C for 15 s, and 40 cycles of denaturation at 95°C for 15 s and annealing for 30 s at 60°C followed by a dissociation step of denaturation at 95°C for 1 min, 55°C for 30 s and 95°C for 30 s to confirm the single specific amplicon.

For all primer sets, the dissociation curve resulted in a single peak and the resulting standard curve showed an *R*^2^ value > 0.9. The primer efficiencies were within 10% difference with the internal control gene. Those gene primers that worked under standard conditions were used further for comparative quantitation using 20 ng of each induced and un-induced sample with the latter referred to as calibrator. The standard thermal cycling conditions were imported to conduct comparative quantitation assays. Non-template controls were included for each reaction, and they showed no amplification and had no Ct value. The data were analyzed using MxPro Software which follows standard Pfaffl (2001) method based on the gene primer efficiencies. All gene expression data were analyzed for relative fold changes using *AtTIP*41 (At4g34270—Czechowski et al., 2005; Han et al., 2013) as normalizer or internal control gene run within each plate for both 24 and 48 h induced and un-induced samples. The fold changes in the induced samples were calculated in the comparative quantitation assay based on the calibrator average. Statistical comparison between induced and un-induced samples was done using one-way ANOVA and Tukey's test for comparison between the induced and the un-induced plants. Analyses were done using SYSTAT Version 10.2 for Windows; significant difference between treatment and control were analyzed at *P* ≤ 0.05. However, only differences that were greater than 2-fold are marked with an ^*^ in the Figures.

## Results

### Increased polyamine biosynthesis draws extra nitrogen and carbon in young seedlings

Since Orn is a limiting metabolite for continued production of Put by the transgenic m*ODC*, and since it is present in relatively small concentrations, and is largely produced from Glu whose biosynthesis requires the continued supply/assimilation of N and C, two specific questions were addressed: (1) Will increased supply of N or C in the growth medium allow extra Put to be produced in the WT and/or the transgenic plants? (2) Will the increased availability of either N or C in the medium affect the amino acids pool in the WT and/or the transgenic plants?

**Figure 2:**
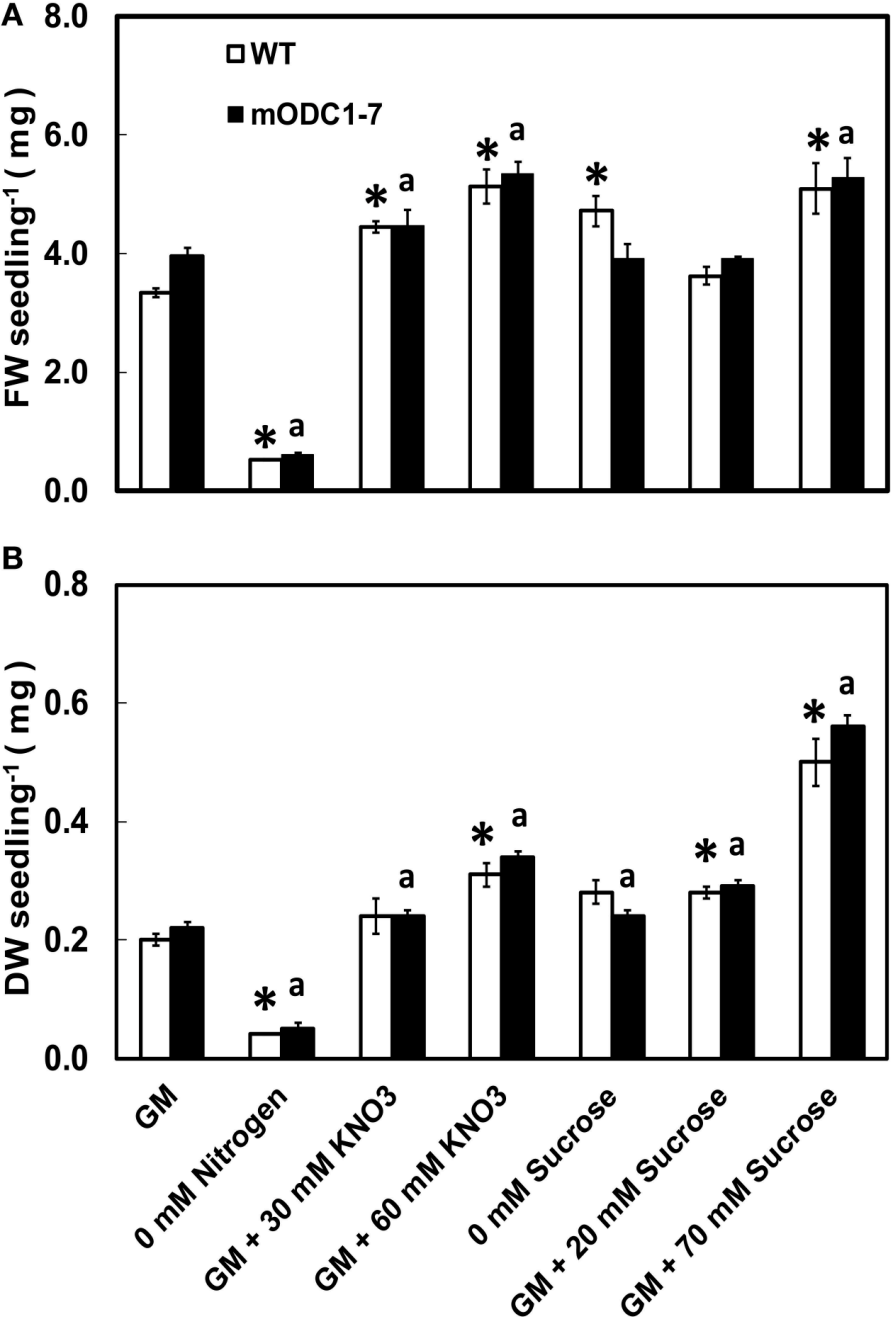
**Effects of different concentrations of nitrate and sucrose on (A) fresh weight (FW) and (B) dry weight (DW) of 12 day old WT and 2x*35S*::m*ODC*-1-7 transgenic *Arabidopsis thaliana* seedlings grown on solid GM**. Data are mean of 4 ± SE; each replicate consists of 40–50 seedlings. ^*^Denotes significant difference between treatments and control in WT seedlings and ^*a*^denotes significant difference between treatments and control in m*ODC* seedlings (*P* ≤ 0.05).

With a few exceptions, the FW as well as DW of seedlings of both genotypes were higher in the presence of additional NO_3_ or sucrose in the medium (over and above the normal concentration in the GM) as compared to those growing in control GM (Figures 2A,B). Additional sucrose at 70 mM had the greatest effect on DW in both genotypes (Figure 2B). Absence of N in the medium caused a significant reduction (vs. GM) in FW and DW of both genotypes.

**Figure 3:**
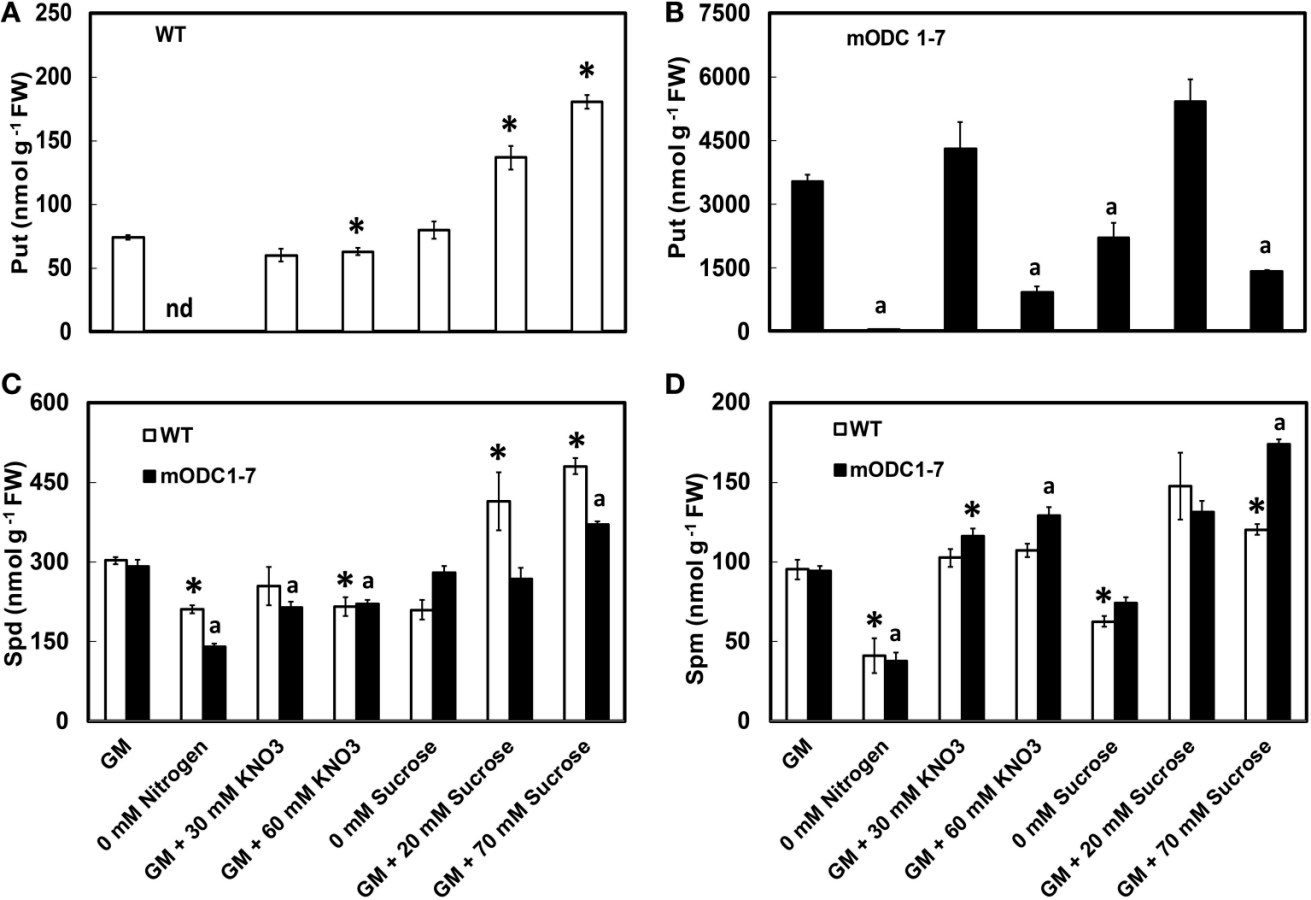
**Effects of different concentrations of nitrate and sucrose on PCA soluble polyamines in 12 day old WT and 2x*35S*::m*ODC Arabidopsis thaliana* seedlings grown on solid GM**. Cellular concentrations of **(A,B)** putrescine, **(C)** spermidine, and **(D)** spermine. Data are mean of 4 ± SE; each replicate = 7–8 seedlings. For symbols, see Figure 2.

When compared to the WT, the m*ODC*-1-7 transgenic seedlings had up to 50 fold higher concentration of Put (Figures 3A,B) with only small changes in the concentrations of Spd and Spm (Figures 3C,D). In the absence of N, Put was reduced by >90% as compared to the control GM for both genotypes, and Spd and Spm were reduced by up to 40%. The seedlings of both genotypes appeared unhealthy (i.e., yellowish) in the absence of N. Supplementation of GM with 30 mM NO_3_ had no effect on Put in either genotype; however, Spd was slightly lower and Spm slightly higher in the transgenic seedlings. At 60 mM additional NO_3_, there was a negative effect on Put and Spd in both genotypes but a small increase in Spm was observed in the transgenic seedlings; the effect on Put was more pronounced in transgenic plants (Figure 3D).

In the absence of sucrose, concentrations of the three PAs were affected differently; the differences were not always significant (Figure 3). In the WT seedlings, additional sucrose caused a concentration-dependent increase in Put, but in the transgenic plants the greatest increase was in response to 20 mM additional sucrose; 70 mM additional sucrose actually having a deleterious effect on Put in these plants. Additional sucrose caused a significant increase in Spd concentration in the WT plants, but less so in the transgenic plants; Spm was higher than the control GM in the transgenic plants given 70 mM additional sucrose.

**Figure 4:**
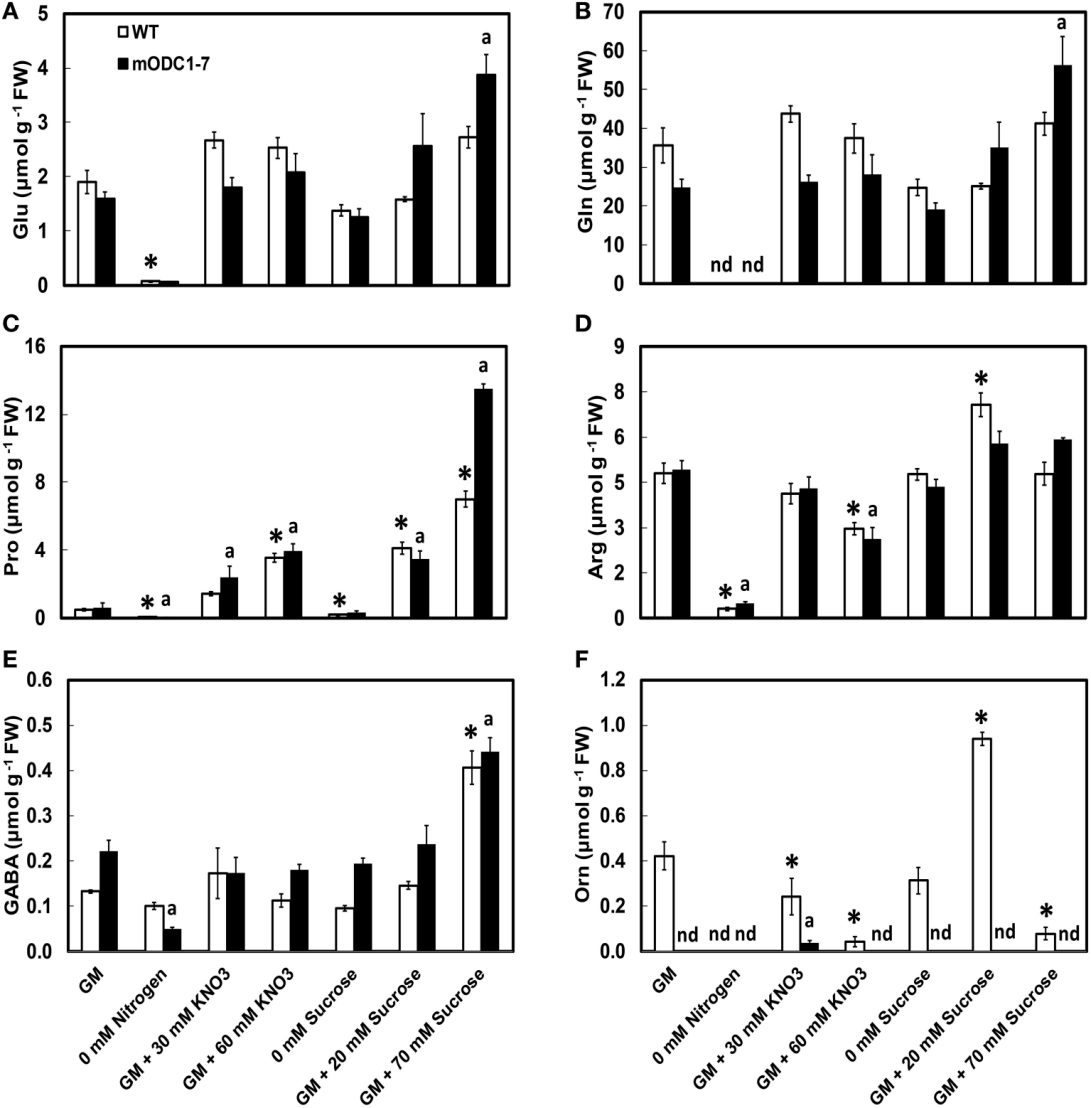
**Effects of different concentrations of nitrate and sucrose on PCA soluble amino acids in 12 day old WT and 2x*35S*::m*ODC*-1-7 *Arabidopsis thaliana* seedlings**. Cellular concentrations of **(A)** glutamate, **(B)** glutamine, **(C)** proline, **(D)** arginine+threonine+glycine, **(E)** γ-aminobutyric acid, and **(F)** ornithine. Data are mean of 4 ± SE; each replicate = 7–8 seedlings. For symbols, see Figure 2.

Significant differences were observed in cellular concentrations of several amino acids in the two genotypes in response to different NO_3_ and sucrose treatments. The abundant (>500 nmol g^−1^ FW) amino acids in both genotypes included Glu, Gln, Arg (+Thr+Gly), serine (Ser), alanine (Ala), and Pro; those present in the group of relatively low concentrations (<100 nmol g^−1^ FW) were Leu, Met, Lys, His, and Trp (Figure 4 and Supplemental Figure 1). Ornithine, which was always present in the WT plants, was below detection limits in the transgenic plants under almost all conditions (Figure 4F). As mentioned above, plants in the N-free medium appeared unhealthy and their cellular amino acid concentrations were highly reduced.

There was no significant difference in the cellular concentrations of Glu or Gln in either genotype in response to additional N or sucrose with the exceptions of increase in both these amino acids in the transgenic plants grown on GM with 70 mM additional sucrose (Figures 4A,B). While the absence of N caused a significant reduction in the two amino acids in both genotypes, the absence of sucrose had no such effect. In the control GM, Pro concentration was similar in the two genotypes and its cellular concentration increased with increasing concentrations of both NO_3_ and sucrose in the medium. In response to 70 mM additional sucrose, Pro concentrations were almost twice in the m*ODC* seedlings vs. the WT seedlings (Figure 4C). The absence of either N or sucrose in the medium significantly reduced Pro in both genotypes.

The combined concentrations of Arg+Thr+Gly (peaks inseparable in most cases by the HPLC method used) were similar in the WT and transgenic seedlings. This group of amino acids was generally unaffected by additional sucrose and both the absence of N as well as additional N at 60 mM caused a reduction in these amino acids (Figure 4D). Whereas, the absence of N resulted in lower cellular concentration of GABA in the transgenic plants, the absence of sucrose had little effect on GABA. Higher sucrose resulted in elevated GABA concentrations in both genotypes (Figure 4E).

In the transgenic plants, Orn was below the detection limit of our technique (Minocha and Long, 2004) in all treatments except for supplementation of the medium with 30 mM NO_3_ (Figure 4F). In the WT plants, higher NO_3_ in the medium resulted in a decrease in Orn concentration. Extra sucrose (GM + 20 mM) caused an increase, but +70 mM caused a decrease in Orn in the WT plants; there was no detectable effect of increased sucrose on Orn in the transgenic plants.

Changes in cellular concentrations of other amino acids that are not direct intermediates/products of the Glu → Orn → Arg/Pro/Put pathway are described in the Supplemental Material section (Supplemental Figure 1).

### Constitutive expression of m*ODC* in mature plants

**Figure 5:**
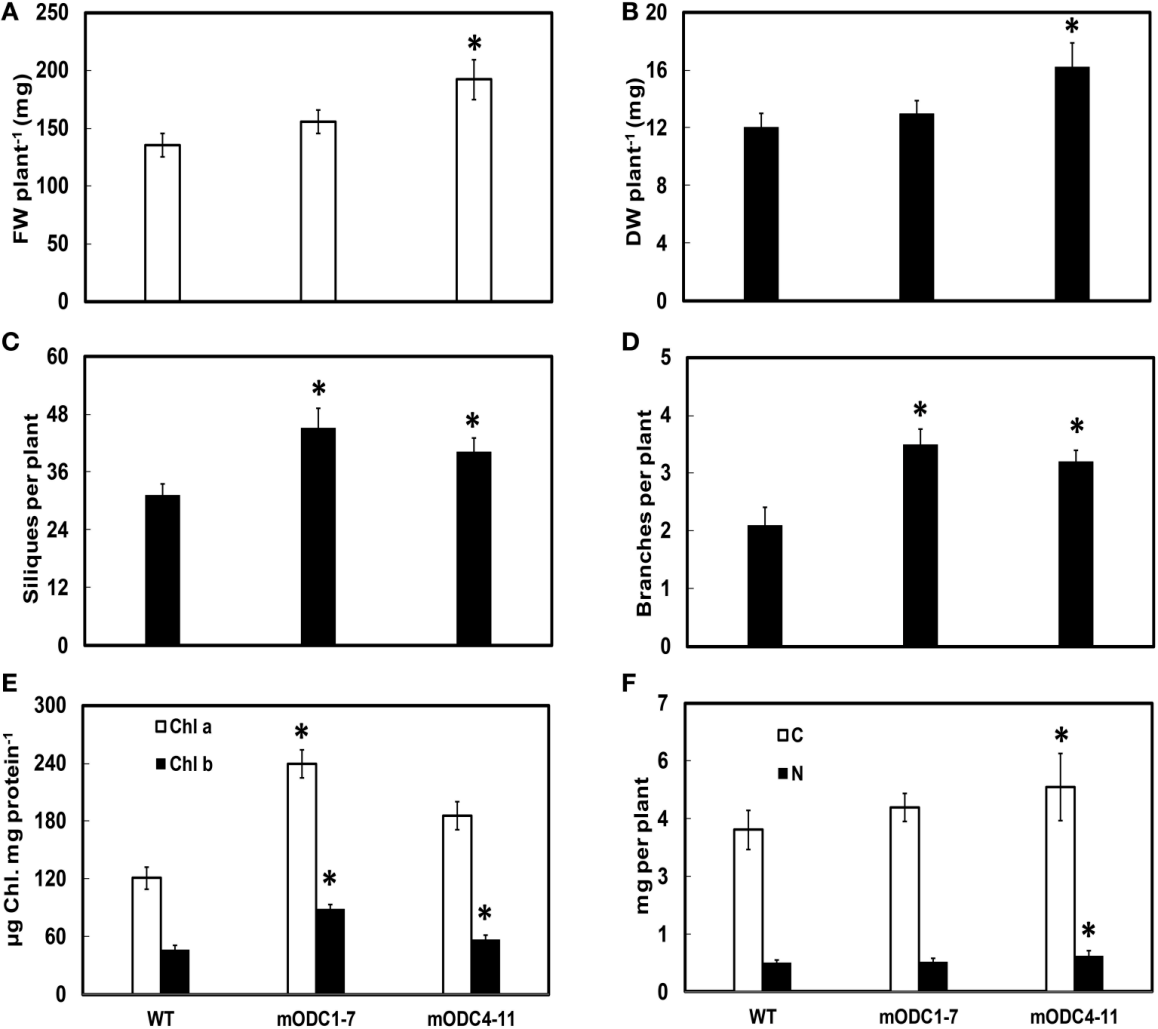
**Comparison of different phenotypic and related biochemical parameters between WT and m*ODC* transgenic *Arabidopsis thaliana* plants for (A) fresh weight (*n* = 9), (B) dry weight (*n* = 9), (C) silique number (*n* = 7), (D) number of branches (*n* = 10), (E) leaf chlorophyll concentration (*n* = 4), and (F) total carbon and nitrogen concentrations (*n* = 10)**. Data are mean ± SE. Plants for **(A–E)** were 6 week-old and near full maturity. ^*^Denotes significant difference between WT and m*ODC* transgenic plants (*P* ≤ 0.05).

Two of the transgenic m*ODC* lines (m*ODC*1-7 and m*ODC*4-11; constitutive expression), which were tested for Put at the seedling stage (Majumdar et al., 2013), showed a delay (generally 7–8 days) in flowering as compared to the WT plants (data not shown). While ~90% of the WT plants were flowering by the end of 5 weeks, <50% of the (m*ODC* constitutive) transgenic plants were flowering at that time. The initial delay in flowering was eventually overcome in the course of time and the transgenic lines showed a significant increase in vegetative as well as reproductive growth. Both FW and DW per plant (at 6 weeks post germination) were significantly higher in the transgenic line m*ODC*4-11 (Figures 5A,B). At full maturity, the number of branches and the number of siliques per plant were greater in both transgenic lines vs. the WT plants (Figures 5C,D).

As compared to the WT, both m*ODC* transgenic lines had significantly higher chlorophyll a and chlorophyll b concentrations on per mg protein basis (Figure 5E). The contents of both total C and total N were slightly, yet significantly, higher in m*ODC*-4-11 plants vs. the WT plants (Figure 5F).

**Figure 6:**
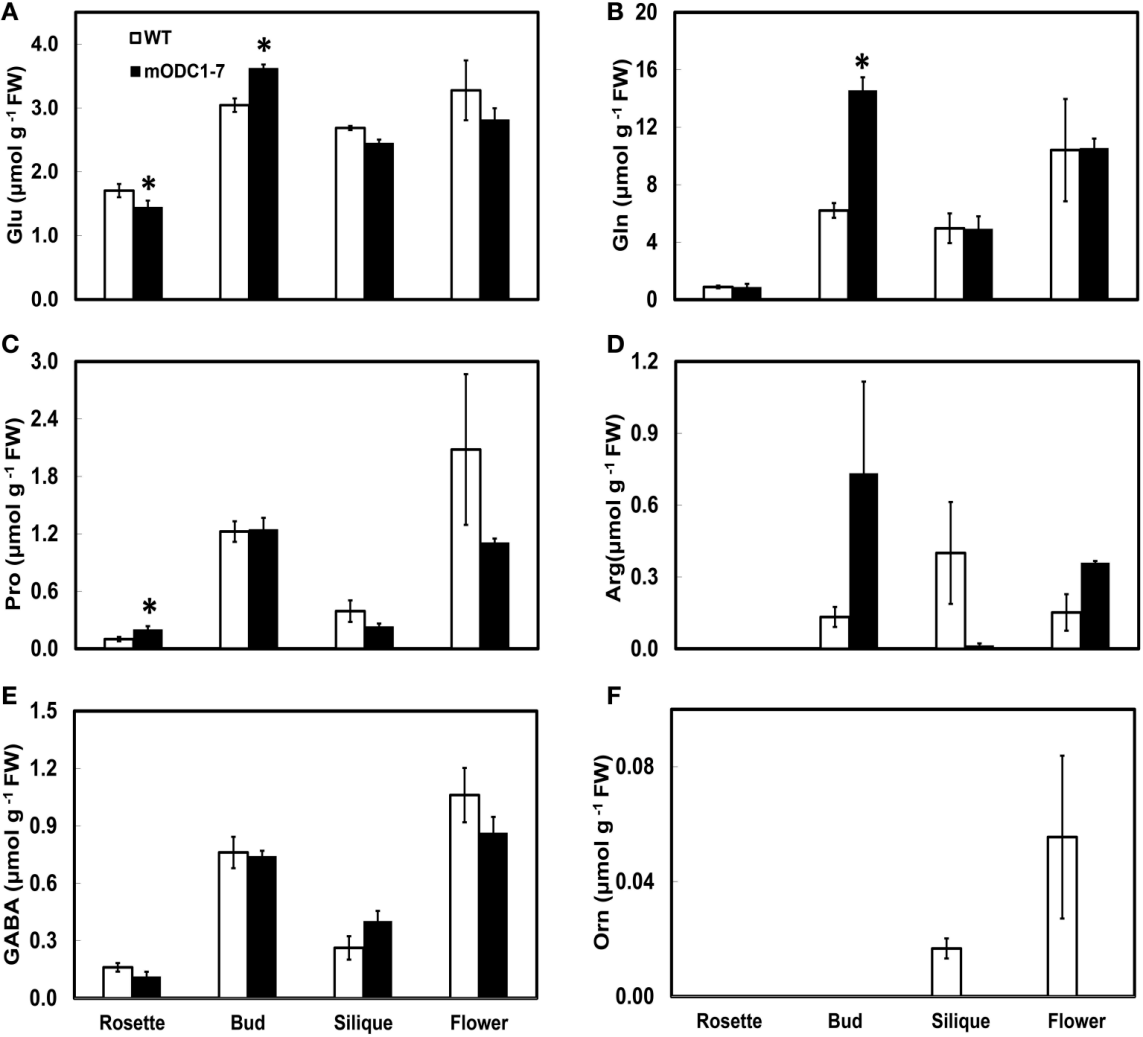
**Cellular concentrations of amino acids (A) Glu, (B) Gln, (C) Pro, (D) Arg, (E) GABA, and (F) Orn, in different tissues of mature (6 week-old) WT and 2x*35S*::m*ODC*-1-7 transgenic *Arabidopsis thaliana* plants**. Data are mean of 3± SE; each replicate consists of 2–3 individual plants. ^*^Denotes significant difference between WT and m*ODC* transgenic plants for each particular organ (*P* ≤ 0.05).

Since the m*ODC*-1-7 line had consistently higher concentration of Put vs. the m*ODC*-4-11 line at the seedling stage, the plants of this line were grown to maturity and various plant organs were analyzed for their PA and amino acid concentrations (Figure 6; Supplemental Figures 2, 3). Significantly higher Put was observed in the cauline leaves and silique tissues of the transgenic plants as compared to the WT plants (Supplemental Figure 2A). The only notable difference in Spd was in the rosette leaves, which was significantly lower in the m*ODC* plants (Supplemental Figure 2B). Spermine was higher in the rosette leaves, buds and flowers of the m*ODC* line as compared with the WT plants (Supplemental Figure 2C). Cadaverine was always present in all organs of transgenic plants, but was never detected in the WT plants (Supplemental Figure 2D).

In mature plants the amino acids of Glu family, whose concentrations were higher in the transgenic plants (vs. WT plants) were Glu and Gln in buds, and Pro in rosette leaves (Figures 6A–C). Conversely, Glu in the leaves, Orn in the flowers, and Arg and Orn in the siliques were somewhat higher in the WT vs. the transgenic plants (Figures 6A,D,F). In all plant parts tested, GABA was not significantly different in the two genotypes (Figure 6E). The remaining amino acids are discussed in the Supplemental Material (Supplemental Figure 3).

### Effect of foliar nitrogen application on polyamines and biomass

**Figure 7:**
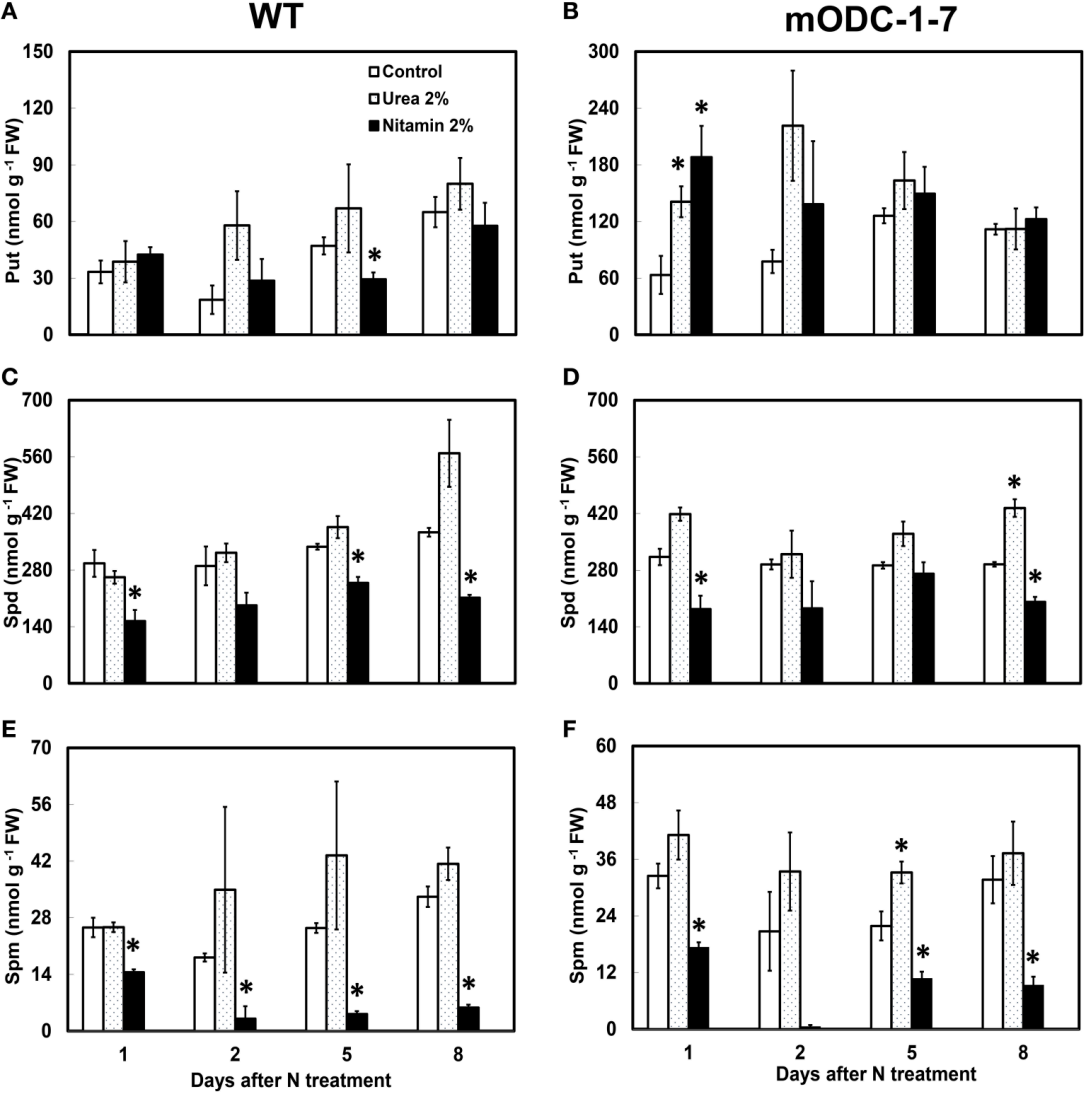
**Effect of foliar nitrogen treatments on PCA soluble polyamines in the rosette leaves of 6 week-old WT and 2x*35S*::m*ODC Arabidopsis thaliana* plants**. Cellular concentrations of **(A,B)** putrescine, **(C,D)** spermidine, and **(E,F)** spermine. Data are mean of 3± SE. ^*^Denotes significant difference between treatment and the corresponding control for each time period for WT and for m*ODC* transgenic plants (*P* ≤ 0.05).

In order to investigate if mature plants producing constitutively high Put were able to assimilate leaf-applied N, WT and constitutive transgenic plants (m*ODC*-1-7) were sprayed with either 2% urea (20:0:0) or 2% Nitamin (30:0:0), and tested for their PA concentration in the rosette leaves, and also for total above-ground plant biomass. As compared to the water sprayed (control) plants, the application of either urea or Nitamin to the foliage resulted in a 2.5 to 3-fold increase in cellular Put within 1 to 2 days after spray in the leaves of transgenic plants (Figures 7A,B); this increase was not sustained after 2 days. On the other hand, there was no significant change in Put concentration in WT plants in response to N treatments as compared to the control (water) treatment. It should be noted that Put concentration of mature leaves of transgenic plants was only 2 to 3-fold higher than the WT plants, whereas at the seedling stage, the transgenic plants often accumulated as much as 40-fold higher concentrations of Put (Figures 3, 7). Cellular concentrations of Spd and Spm were similar in the WT and the transgenic plants, and generally decreased at 24 h in response to Nitamin treatments (Figures 7C–F). Nitamin treatment caused a significant decrease in Spm in both genotypes at all times of analysis. There was no significant increase in DW of transgenic plants in response to foliar N application (Supplemental Figure 4).

### Gene expression of the Glu-Orn-Pro/Arg-PA-GABA pathway enzymes in response to increased put biosynthesis

**Figure 8:**
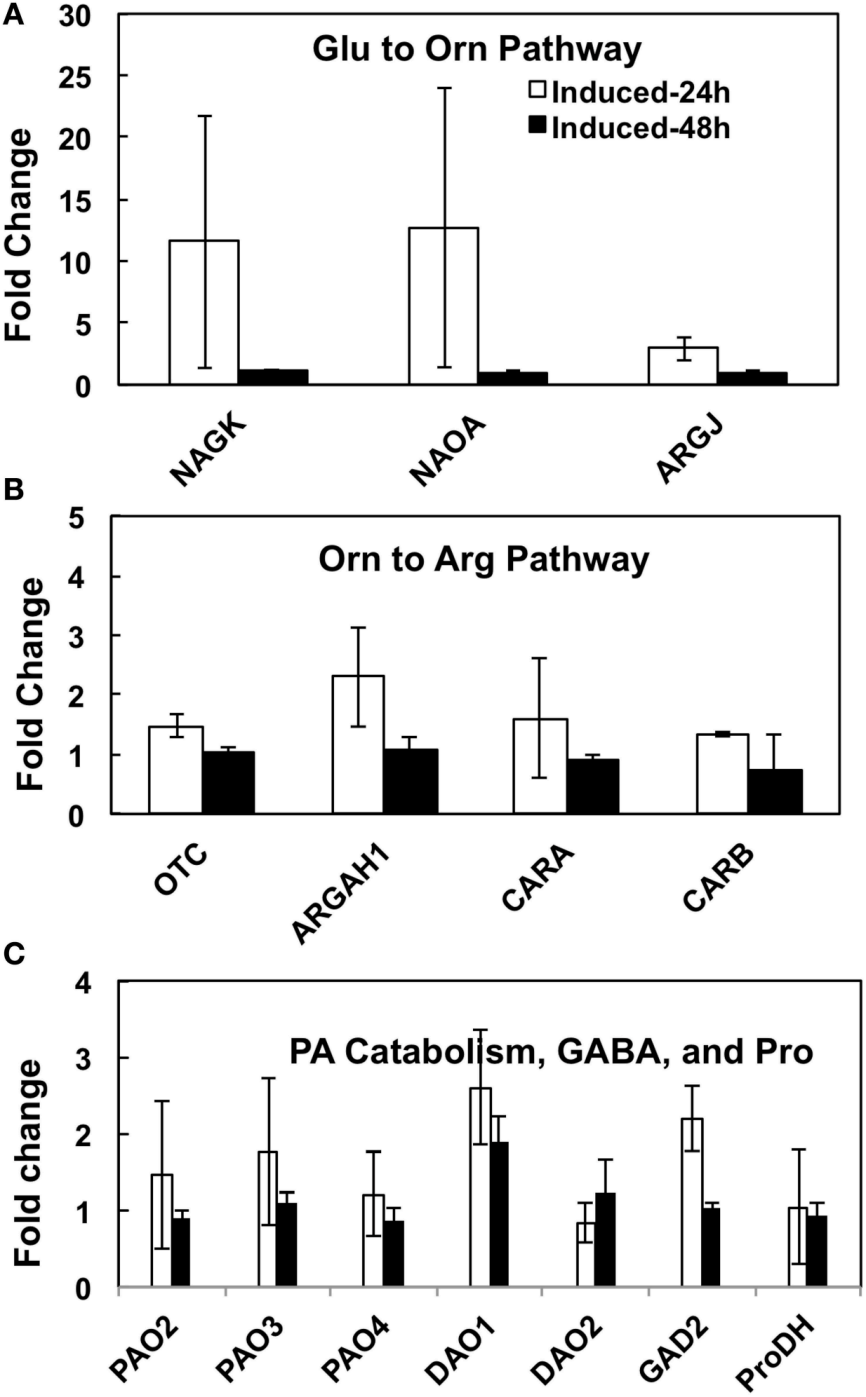
**The effects of inducible increase in putrescine production on changes in the relative expression of various genes of (A) Glu to Orn, (B) Orn to Arg, and (C) PA catabolism and GABA biosynthesis pathways in *Arabidopsis thaliana***. cDNA from transgenic m*ODC* induced and un-induced seedlings collected at 24 and 48 h after induction with estradiol were used with gene specific primers for qPCR. The qPCR assays were conducted with two technical replicates per biological replicate (see details in Supplemental Table 2). Data are Mean ± SE of 3 biological replicates. For explanation of enzymes names refer to legend to Figure 1 and for gene names refer to Supplemental Table 1. The bars represent fold change in gene expression in the m*ODC* induced as compared to the un-induced control seedlings. ^*^Denotes difference between m*ODC* induced seedlings and the corresponding un-induced control plants at the same time of analysis where the difference is significant (*P* ≤ 0.05) and at least 2 fold; differences that were significant but ≤2 fold are not shown.

Our experimental manipulation of one step (i.e., Orn → Put) in the interactive pathways shown in Figure 1, using a transgene that has no sequence homology to a native gene in *A. thaliana*, obviously resulted in overutilization of Orn, which must be replenished via its increased biosynthesis from Glu to serve the demands of m*ODC* as well as the other products of these pathways. The pathways of Glu to Arg, Pro, PAs, and GABA involve about 20 enzymes encoded by >30 genes that have been identified in *A. thaliana* (Supplemental Table 1). In order to understand the transcriptional regulation of Glu → Orn → Arg, Glu⇔Pro⇔Orn, Put/PA catabolism, and Glu → GABA in response to short term (24–48 h) increase in Put via induction (by estradiol) of the inducible m*ODC* transgene, we addressed three complementary questions in the present study: (i) Does the increased biosynthesis of Orn from Glu require the induction of genes encoding various enzymes involved in Orn biosynthesis? (ii) Does the overutilization of Orn affect gene expression of the enzymes involved in the interacting pathways of Arg, Pro and GABA biosynthesis? (iii) Does the expression of genes involved in PA catabolism (some that produce GABA) change in response to overproduction of Put? The technique of qPCR using primers specific for each of the known *A. thaliana* genes of these sub-pathways was used with cDNA made from mRNA isolated at 24 and 48 h after the induction of m*ODC* in young seedlings. The results presented in Figure 8 and Supplemental Figure 5 are the results of two separate experiments using the same inducible transgenic cell line but slightly different approaches for qPCR setup and analyses of results (See Materials and Methods, Figure legends and Supplemental Material for details). The results of relative expression of various genes are grouped according to sub-pathways of Glu → Orn, Orn → Arg, Glu⇔Pro⇔Orn, Put/PA catabolism and Glu → GABA production.

The m*ODC* transgenic seedlings used for qPCR typically showed several-hundred-fold increase in m*ODC* transcripts upon induction, with very little m*ODC* transcript being detected in the un-induced plants (data not shown). There was a concomitant 10- to 20-fold increase in Put in the induced seedlings vs. the control (un-induced) seedlings at 24 and 48 h, with only small or no change in Spd and Spm (data published earlier - Majumdar et al., 2013).

### Glutamate to Orn-Arg-Pro pathway genes

Of the six enzymes involved in Glu to Orn subpathway, cDNA sequences for all genes except NAOD were available in the literature (Supplemental Table 1). Six of the seven total genes were tested for change in their relative expression (vs. un-induced) following induction at 24 and/or 48 h; none showed a significant change that was greater than 2-fold (Figure 8A and Supplemental Figure 5A). Only one gene of the Orn → Arg pathway (i.e., CARB) encoding the large subunit of CPS (Gln-dependent carbamoyl-P synthetase) showed a significant increase in relative expression on induction at 24 h in one experiment (Supplemental Figure 5B); a repeat of this experiment did not show such a large change in expression at either 24 or 48 h of induction (Supplemental Table 2, Figure 8B).

There are seven genes that encode for five enzymes involved in Glu⇔Pro⇔Orn interconversion (Supplemental Table 1); the expression of all of these genes was similar in the induced and un-induced plants at 24 as well as 48 h (Figure 8C and Supplemental Figure 5C). The gene *P5CR* has two splice variants in *A. thaliana* - *P5CR* - NM_001085115.1, and *P5CR*.1 - NM_121484.4; the primers designed for qPCR (Supplemental Table 1) were able to distinguish between the two splice variants.

### Genes of polyamine catabolism and GABA biosynthesis related genes

Of the five *PAO* and two *DAO* genes involved in PA catabolism, none showed a consistent change greater than 2-fold on induction of m*ODC* (Figure 8C, Supplemental Table 2 and Supplemental Figure 5D). Only one gene for Glu catabolism into GABA (*GAD*2) was successfully amplified (others showed multiple bands in endpoint PCR); its expression was not different in the induced plants vs. the un-induced control.

## Discussion

Understanding the regulation of PA and amino acid metabolism in plants is of major interest (Sinclair et al., 2004; Slocum, 2005; Kalamaki et al., 2009; Rees et al., 2009) because these two groups of metabolites occupy key positions in connecting N metabolism, C fixation, and several pathways associated with secondary metabolism. In this regard, Orn is a key metabolite sitting at the crossroads of several interactive pathways involving major amino acids: Glu → Orn → Arg → Put, Glu → Orn → Pro, Arg → Orn → Glu and Pro (Figure 1). In earlier reports (Majumdar et al., 2013, 2015; Minocha et al., 2014), we have argued that adding the transgenic m*ODC* shunt in *A. thaliana* would significantly disturb the homeostatic flux of Glu to PAs, Pro, Arg and GABA by diverting large amounts of Orn (the substrate for m*ODC* as well these metabolites) to the production of Put. Further, it was postulated that increased metabolic conversion of Glu/Orn into Put may considerably affect the pool of other amino acids in the cell, thus leading to N deficiency and decrease in protein synthesis.

For this study, we postulated that in the m*ODC* transgenic plants, the depletion of Orn caused by its increased use by m*ODC* will result in increased conversion of Glu to Orn, which would be compensated partially by increased biosynthesis of Glu from assimilated N and C. Furthermore, we surmised that the production of Pro may be affected by increased utilization of Glu into Orn/Arg part of the pathway.

Glutamate can be considered as the “Center of the Universe” for N metabolism in plants since most of the assimilated N passes through this step before it is re-distributed to major N metabolites (reviewed by Forde and Lea, 2007). While, it is the primary source of amide group for all protein amino acids via transamination reactions, it is also a direct precursor of several other N-rich metabolites, which play important physiological roles in plant development and stress response; PAs and GABA being among them (Minocha et al., 2014; Majumdar et al., 2015). Furthermore, Pro and Arg, for which Glu is the direct source in plants, also play important roles in stress response (reviewed in Minocha et al., 2014; Majumdar et al., 2015) and in the production of signal molecules like NO (Morris, 2007, 2009). Thus, the distribution of Glu into different competing but complementary pathways must be intricately regulated to achieve homeostatic levels of various products whose biosynthesis depends on this amino acid. At the same time, it is well established that overall N assimilation in plants has a complex dependence on the availability of C and *vice versa*. It is therefore not surprising that major rechanneling of N has wide-ranging effects on the reallocation of cellular C. Under conditions of “threat to survival” (e.g., abiotic stress due to non-toxic conditions/treatments), the plants respond by reallocating N as well as C into metabolites like Pro, GABA, PAs, glycinebetaine and β-Ala (together referred to as compatible solutes), partly because they play protective roles under stress and perhaps also help alleviate toxicity of NH_3_ produced within the cells. The Glu-Pro-Arg-PA-GABA pathway is thus central to the biosynthesis of these metabolites to achieve a balance between assimilated N and C in the plant. In the present study we explored wider effects of altering the flow of Glu to PAs by creating a shunt for redirecting Orn toward Put.

### Ornithine as a gatekeeper in controlling PAs, Pro, and GABA biosynthesis

In plants, Orn is usually present in very small concentrations as compared to its products Arg, Pro, and Glu; its biosynthesis from Glu involves several enzymes (Shargool et al., 1988; Slocum, 2005; Figure 1). The first (regulatory?) step in Orn biosynthesis in plants is believed to be Glu → NAG via NAGS; this is in contrast to animals where nutritional Arg is the primary source of Orn, and the reaction is controlled by arginase (Morris, 2006, 2007). Morris (2009) has argued for the role of charged tRNA^Arg^ in regulating cellular Arg levels in mammals. As an extension of the argument of Ramos et al. (1970) about the role of Orn and citrulline (Cit) in regulating Arg-Orn pathway, we argue in favor of the importance of cellular Orn in regulating the Glu → Orn flux, particularly in response to increased demand (or depletion) of Orn by m*ODC* in the transgenic plants. It was shown earlier in poplar cell cultures that this increased flux apparently does not involve increased expression of most of the genes encoding enzymes of this pathway, except perhaps NAGS (Page et al., 2012).

Kalamaki et al. (2009) demonstrated that constitutive over-expression of a tomato *NAGS*1 gene in Arabidopsis led to higher accumulation of Orn and Cit in leaves without significant increase in Arg concentration. In the present study, we contend that significant changes in protein- and non-protein amino acids that accompany the increase in Put via m*ODC* shunt must cause increased flux of Glu → Orn proportionate to its demand imposed by the transgenic m*ODC*. Additionally, since Orn biosynthesis is distributed in several cellular compartments (Shargool et al., 1988) but its consumption by m*ODC* is entirely cytoplasmic, it can be argued that Orn transport across organelles must also be co-regulated with its consumption in order to ensure its continued availability for its multiple usage. Another interesting aspect of Orn metabolism revealed by this study is that the amount of Put accumulating in mature transgenic plants was several-fold lower than that in the corresponding seedlings. This suggests two possibilities: either the m*ODC* production (transcription or translation) was lower in mature tissues, or the flux of Glu → Orn/Arg or its transport to the cytoplasm was low for m*ODC* to work optimally. This is consistent with the presence (in mature plants) of large amounts of Cad, apparently the product of m*ODC* using Lys as a substrate.

### γ-aminobutyric acid production from gad and put catabolism

Regulation of GABA metabolism in plants is complex since various enzymes associated with GABA metabolism are spatially compartmentalized in the cell (Shelp et al., 2012b). Whether or not its biosynthesis and catabolism are regulated at the transcription level is not known. Our results on the production of GABA via Put catabolism in m*ODC* transgenic cells of poplar (Quan et al., 2002; Lasanajak et al., 2014) and seedlings of Arabidopsis (data presented here) do not indicate the induction of DAO even though the production of GABA through this pathway is increased several-fold. However, since there are additional *DAO* genes as well as the *GAD* genes that we did not analyze, these conclusions may be tentative. Our findings that higher Put degradation can occur without increase in *DAO* expression are consistent with those of Shelp et al. (2012a). However, the question of relative contributions of the anabolic (GAD) and the catabolic (DAO) pathways for GABA production to maintain its homeostasis in plants still remains unanswered.

The lack of change in Glu concentration in both genotypes by extra NO${}_{3}^{-}$ in the medium with/or without concomitant change in either GABA or PA concentration (Figures 3, 4) are consistent with the suggestion of Fait et al. (2008), who emphasized the role of GABA in repartitioning of C and N during seed development and germination in *GAD* transgenic seedlings of Arabidopsis. In line with these findings, we observed that greater availability of N in the medium could enhance N metabolism and growth (biomass?) in the m*ODC* transgenic plants without C becoming limited. A similar outcome was observed for C supplementation of the medium; however, at higher concentrations of C, N appeared to become limited for Put production in the transgenic plants but not for GABA production. These results strongly suggest that the redirection of Orn into Put and then into GABA shunt could enhance C uptake and assimilation through increased N assimilation, thus restoring Glu loss due to Orn depletion.

### Gene expression and the regulation of Glu → Orn → Arg pathway

Nabais et al. (2005) studied the role of Orn in N remobilization in evergreen *Quercus ilex* and found that in early spring Orn was the first detectable amino acid in the xylem sap (presumably resulting from Arg degradation via arginase). Similar increases in Orn have been reported in germinating seeds when protein reserves are used to generate Glu, Gln, and Pro from Arg (Cañas et al., 2008). This situation is analogous to mammalian Glu and Gln biosynthetic pathways where nutritional Arg is metabolized into other amino acids and PAs, and for NH_3_ detoxification via the combined actions of Arginase and urease (Boon et al., 1999; Levillain et al., 2004). In young Arabidopsis seedlings, the primary source of Orn was Glu (and not Arg), which was produced from assimilation of N from the medium. While OAT is involved in Arg conversion to Glu following Arginase-urease actions, the regulatory enzyme for Orn (and hence Arg and Put) production from Glu is considered to be NAGS (Figure 1). Our results indicate that neither of the two *NAGS* genes was up-regulated on induction of m*ODC*, which is inconsistent with its presumed role as a potential regulatory step in Glu → Orn biosynthesis (Kalamaki et al., 2009). Regulation of the remaining set of reactions involved in Glu → Orn → Arg flux is not known. Our past qPCR analysis with high Put poplar cells did not show major changes in the expression of most genes encoding enzymes of this pathway in poplar (Page et al., 2012); the results with *A. thaliana* presented here further corroborate these observations. Surprisingly, despite several-folds increase in demand for Orn by m*ODC*, there was no major effect on either the expression of genes coding for enzymes of the Orn → Arg or those coding for Glu → Pro parts of the pathway. Concurrently, the resulting large decrease in Glu was compensated at least partly by increased N assimilation into Glu, especially under conditions of abundant N and C in the medium. Unfortunately, the current study did not involve expression of the genes encoding either enzymes of the N assimilation or those involved in C uptake/assimilation pathways. An important conclusion from these results is that a significant proportion of N assimilation is driven by downstream N utilization as long as C is not limiting. Furthermore, it can be argued that the limitation of C under these conditions may also be alleviated (at least partially) by its enhanced uptake and assimilation from the medium. Whether or not such an increase in the metabolic flux of inorganic N into this pathway would lead to increase in C fixation through photosynthesis has not yet been tested in mature transgenic plants.

### Regulation of Glu⇔Pro⇔Arg

Sucrose concurrently affected Pro and GABA (both being products of Glu), but the responses were different in the transgenic vs. the WT seedlings. The results suggest that in the transgenic plants, re-routing of Glu into PAs might have caused a limitation of N for Pro production, which was reversed by additional N in the medium. Based on observations that limitation of C in the medium affected Pro, Arg, and GABA, and the addition of sucrose to the medium enhanced the accumulation of all three of these amino acids, it can be argued that: (1) the N reserves of transgenic cells producing high Put are more rapidly diluted than the C reserves, and (2) its reversal by adding extra N does not lead to the uptake and assimilation of C. However, having extra C available to the plants leads to additional N uptake, a situation if translated in the field grown plants should result in enhancement of total biomass through this increased N use in the plants. The response of WT and high Put transgenic plants to additional C in the medium was much less pronounced for Gln and Arg, indicating a different level of regulation for these two amino acids. Ornithine still remained undetectable in the transgenic plants regardless of the addition of N or C, suggesting that this amino acid was still limited for the transgenic m*ODC*.

Proline biosynthesis from Orn in plants is apparently not regulated by feedback inhibition of the enzymes δ-OAT and P5CR (Larosa et al., 1991). Induction of m*ODC* resulted in up-regulation of Pro (as also seen in high-Put poplar cells by Mohapatra et al. (2010b). Higher accumulation of GABA and Pro both under inducible and constitutive m*ODC* expression conditions in *A. thaliana* seedlings and mature plants indicate that these plants might be useful tools to study a wide range of stress responses. Whereas significant changes in Pro, Glu, GABA and Put were seen in response to additional C, changes in the accumulation of Spd and Spm were relatively small; reconfirming our earlier evidence of an independent and tight regulation of their biosynthesis and accumulation.

### Phenotypic changes in m*ODC* transgenic plants

While the transgenic plants constitutively over-expressing m*ODC* were phenotypically similar to the WT plants, there were subtle developmental differences between the two genotypes. The transgenic plants had higher fresh biomass as well as dry mass per plant just before bolting, which was delayed by at least a week as compared to the WT plants. Higher chlorophyll concentration in the transgenic plants, and greater number of branches and siliques, all seem to indicate positive effects of high Put production in the present study. These effects are consistent with the observed increase in total C and N in the transgenic plants, which may be related to higher chlorophyll (photosynthesis?) in the leaves. The diamine Put has been shown to promote light reactions of photosynthesis through increased photophosphorylation (Ioannidis et al., 2006), and it is also the best stimulator of ATP synthesis as compared to Spd and Spm (Ioannidis and Kotzabasis, 2007). If this turns out to be the case, we would have discovered an alternate way to manipulate photosynthesis via N assimilation into PAs to increase biomass in bioenergy crops.

## Conclusions and future perspectives

Over the years, metabolic engineering in plants has gained considerable attention in transgenic research to enhance the nutritional value of food/feed crops, to improve the abiotic stress tolerance of plants (Hussain et al., 2011; Mattoo et al., 2011), improved quality of flowers and fruits (Lücker et al., 2001; Goff and Klee, 2006), increased production of secondary metabolites (Pilate et al., 2002; Verhoeyen et al., 2002), and increase in essential amino acids (e.g., Lys, Met, Trp) in food and forage crops (Galili and Höfgen, 2002). Various mechanisms have been postulated in controlling metabolic fluxes of connected pathways that share a common substrate (Allen et al., 2009; Palsson, 2009; Grüning et al., 2010). One mechanism of increasing the cellular production/concentration of a metabolite without increases in transcription or translation of the related enzymes can be through increasing the demand for utilization of the product (Zhu and Galili, 2003), i.e., the metabolic pull (or a sink). While most studies have lacked experimental evidence involving detailed analysis of the pleiotropic effects of such metabolic manipulations, several reports from our lab (Page et al., 2007, 2012; Mohapatra et al., 2010a,b; Page et al., unpublished) have demonstrated that manipulation of a single step in the PA biosynthetic pathway (i.e., increased Put production via transgenic *ODC*) can cause a major redirection of the cell's metabolome. Thus, for plants to be used for industrial purposes (e.g., bioenergy, biofuels, pharmaceuticals, enzyme catalysts, etc.), metabolic engineering to increase N assimilation, and the resulting/accompanying C sequestration via manipulation of PA biosynthesis may provide a useful tool in not only producing greater biomass under conditions of optimal growth but also under stress because of the beneficial effects of higher PAs in improving stress response.

Some of the specific conclusions from this study are: (1) pathway from Glu to Orn and from Orn to Arg is regulated primarily at the enzymatic level (vs. gene transcriptional level) and it involves tight regulation of cellular Orn concentration; (2) production of Pro is regulated independently of the Glu → Orn → Arg part of the pathway; and (3) depletion of Glu caused by increased flux toward Orn/Arg and Pro may be compensated partially by its increased biosynthesis from assimilated N.

## Funding

Partial funding was provided by the New Hampshire Agricultural Experiment Station. This is Scientific Contribution Number 2608. This work was supported by the USDA National Institute of Food and Agriculture (McIntire-Stennis) Project (NH00062 and NH00076-M).

### Conflict of interest statement

The authors declare that the research was conducted in the absence of any commercial or financial relationships that could be construed as a potential conflict of interest.
