# Supplementary material for: Glutamate, Ornithine, Arginine, Proline, and Polyamine Metabolic Interactions: The Pathway Is Regulated at the Post-Transcriptional Level
Source: Front Plant Sci. 2016 Feb 16;7:78. doi: 10.3389/fpls.2016.00078 (PMC4754450; doi:10.3389/fpls.2016.00078)
Supplement: Supplementary file 1 [file Presentation1.pdf]

SUPPLEMENTAry MATERIAL - Majumdar et al.

Changes in other amino acids (other than those that are discussed in the main text) in response to extra Nitrogen and Carbon in the medium

I. Concentrations of all amino acids except Ala and Lys were similar in the two genotypes; the former was higher and the latter was lower in the transgenic plants. Most of the amino acids were negatively affected by the absence of N in the medium but not so in the absence of sucrose.

II. Histidine was a minor amino acid in all cases except in the presence of additional N; in that case both WT and transgenic seedlings had similar concentrations of His (Supplemental Figure 1a).

III. The absence of N caused a significant reduction in Ser (a major component of the soluble amino acids fraction), the presence of extra N in the medium had no effect and additional sucrose caused a significant increase in Ser only in the transgenic plants (Supplemental Figure 1c).

IV. In both genotypes, Phe was lower in the absence of N and significantly higher when the medium was supplemented with extra N (Supplemental Figure 1c); 100 mM extra sucrose had a small but significant effect in transgenic plants only.

V. Ala and Val concentrations were positively affected by additional N as well as C, but the response was different in the two genotypes (Supplemental Figures 1e, 1f). While Ala content was significantly higher in the m*ODC* plants than the WT in all treatments; Val showed difference between the two genotypes only in the presence of extra sucrose in the medium. Extra sucrose positively affected Ile in both genotypes (Supplemental Figure 1g).

VI. Cellular concentrations of Met (Supplemental Figure 1i), Lys (Supplemental Figure 1h) and Leu (Supplemental Figure 1d) were generally low in both genotypes, and showed smaller variations in response to the experimental treatments except that Lys was typically lower in the m*ODC* plants.

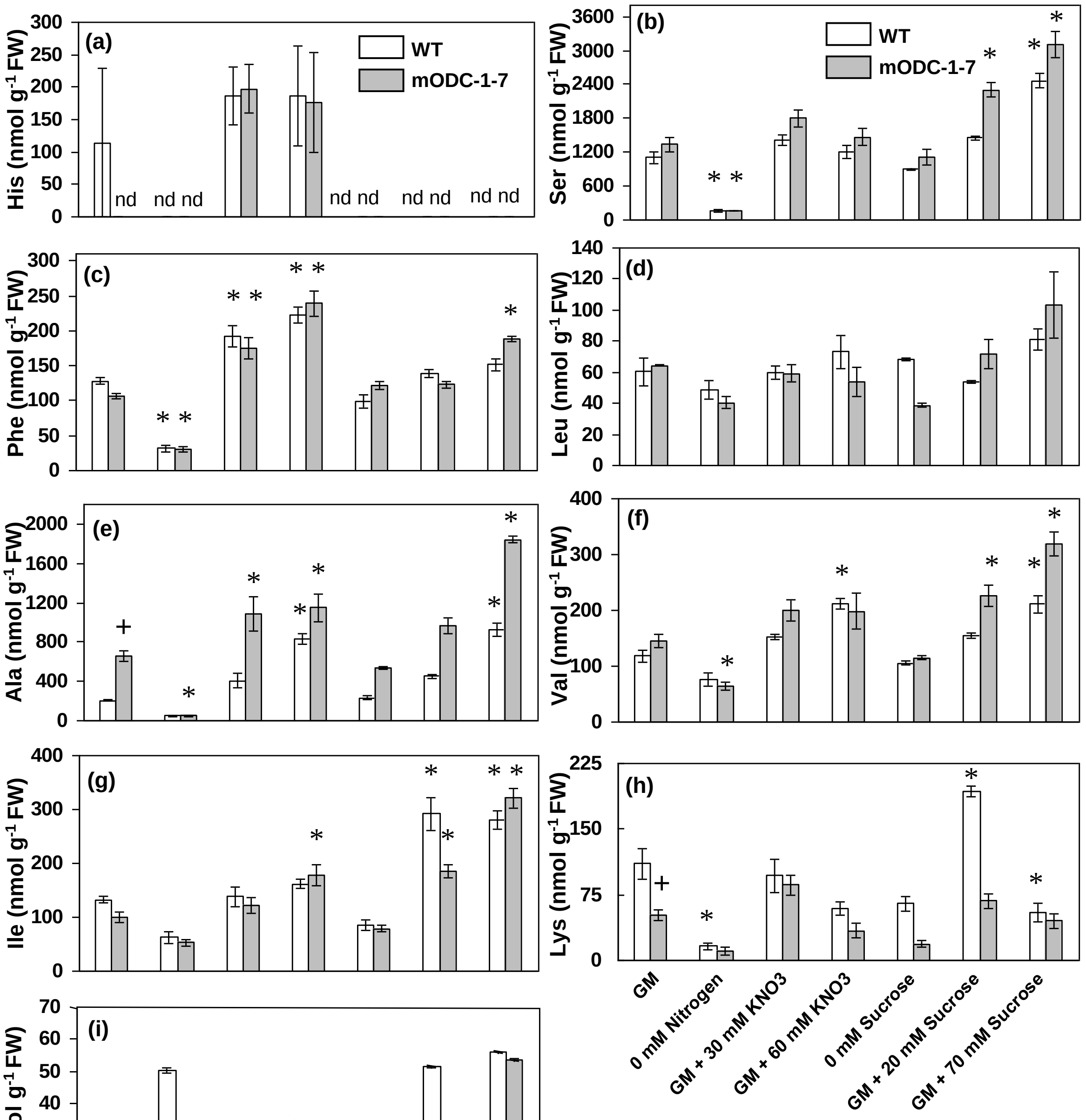

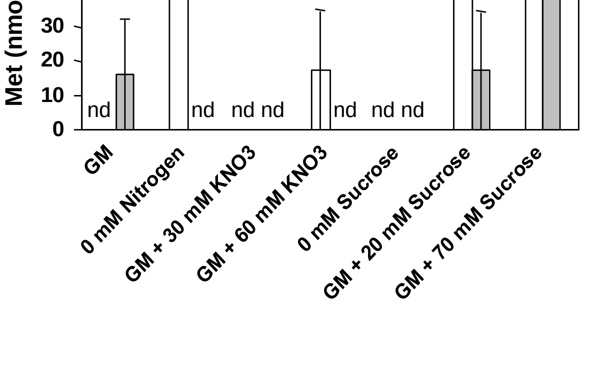

Supplementary Figure 1. Effects of different concentrations of nitrate and sucrose on PCA soluble amino acids in 12 d old WT and m*ODC*-1-7 plants. *= P≤ 0.05 for significant difference between treatments and control within WT and m*ODC* plants; + represents P≤ 0.05 for significant difference between WT and m*ODC* plants (N= 3; each replicate= 7-8 seedlings).

Polyamine Contents in Mature Plant Parts:

I. Whereas the cauline leaves and siliques showed 2 to 3-fold higher concentrations of Put in the transgenic plants (Supplemental Figure 2a), rosette leaves, flower buds and mature flowers did not show significant differences in their Put contents vs. the WT counterparts.

II. Spermidine concentrations followed a similar trend as Put in various organs and were quite similar in the respective organs of the two genotypes (Supplemental Figure 2b).

III. Like Put, in most tissues, Spm was higher in the transgenic plants (vs. the WT) in several organs (Supplemental Figure 2c).

IV. All of the mature organs that were tested contained significant amounts of Cad, which is presumably derived from Lys by the activity of mODC (Supplemental Figure 2d); WT plants had no Cad in any organ.

V. Among the different organs, flower buds and mature flowers typically had the highest concentrations of all four PAs (Supplemental Figure 2e).

Amino Acids in Mature Plant Organs

I. In mature plants, the amino acids whose concentrations were significantly higher in transgenic vs. the WT plants (excluding those mentioned in Figure 4) in different organs included His, Leu, Phe, Leu, and Val in the buds (Supplementary Figure 3).

II. Conversely, Thr in the leaves, Ala in the buds, and His in the siliques were somewhat higher in the WT than in the transgenic plants.

III. In general, total soluble amino acids were higher in the buds and the flowers in both the transgenic and the WT plants. No significant difference in total soluble amino acids was observed among other organs except for the buds of m*ODC* plants that showed significantly higher amount of total soluble amino acids than the WT buds.

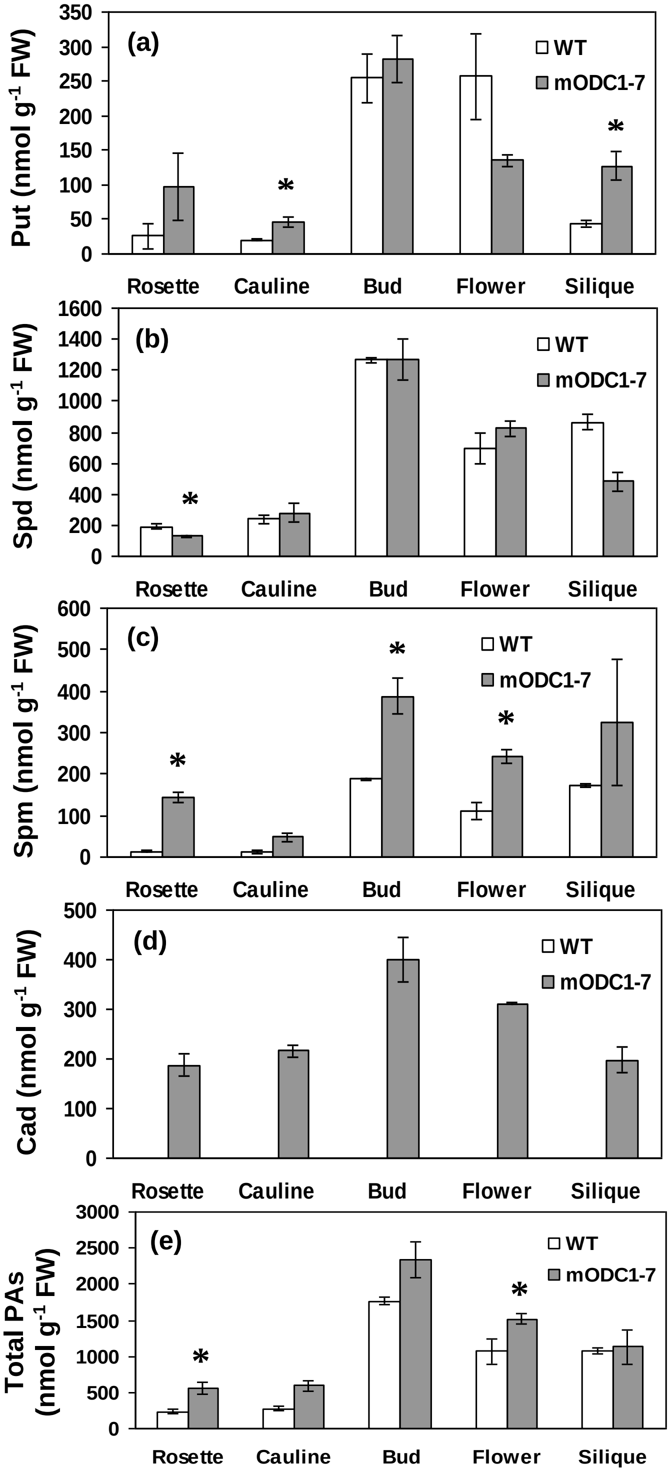

Supplementary Figure 2. Cellular contents of PCA soluble polyamines in different organs of 6 week-old WT and 2x*35S*::m*ODC*-1-7 transgenic plants. *= P≤0.05 for significant difference between two genotypes (N= 3; each replicate= tissue obtained from 2-3 individual plants).

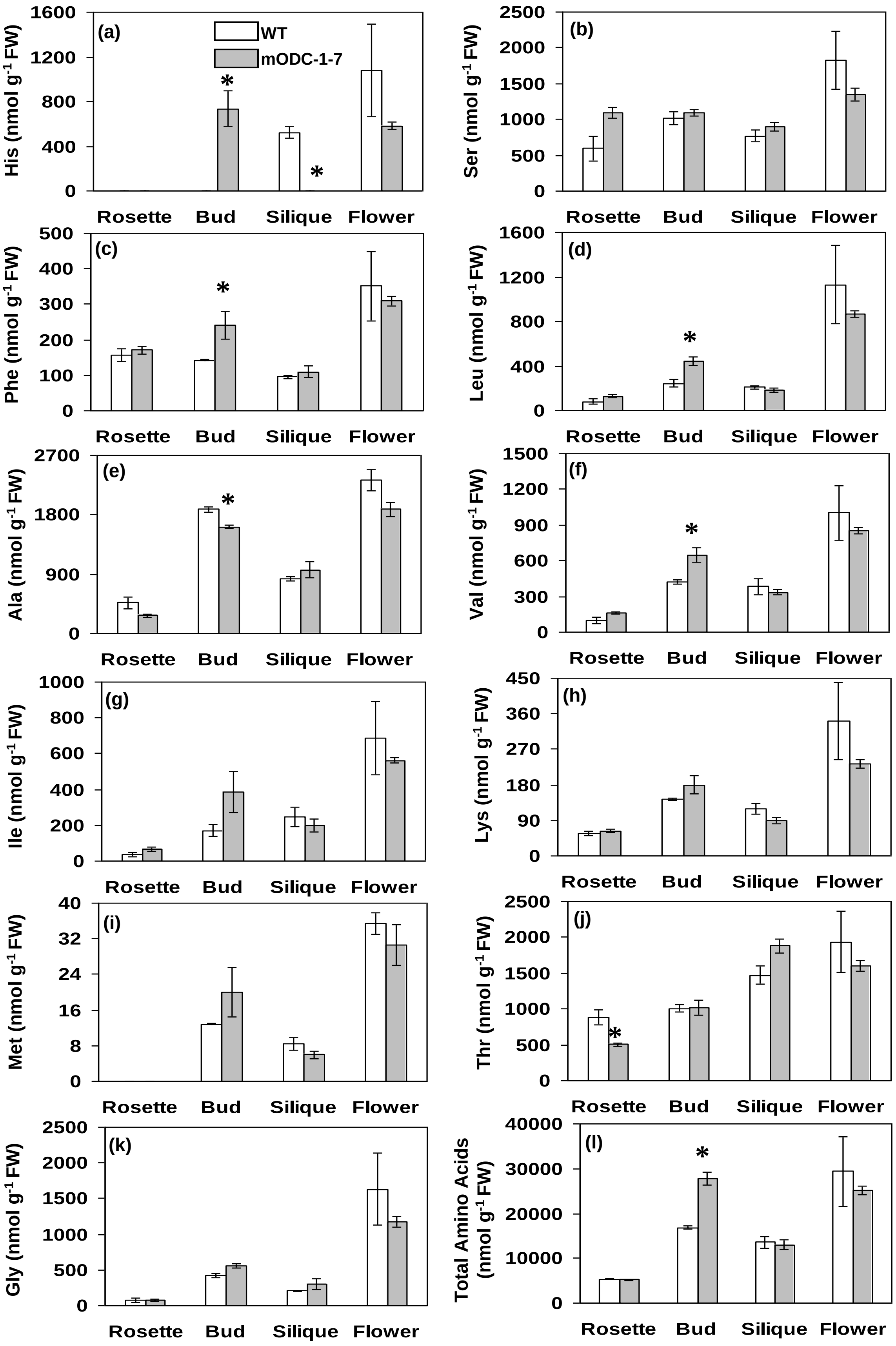

(a)
WT
mODC-1-7
His (nmol g-1 FW)
(b)
Ser (nmol g-1 FW)
(c)
Phe (nmol g-1 FW)
(d)
Leu (nmol g-1 FW)
(e)
Ala (nmol g-1 FW)
(f)
Val (nmol g-1 FW)
(g)
Ile (nmol g-1 FW)
(h)
Lys (nmol g-1 FW)
(i)
Met (nmol g-1 FW)
(j)
Thr (nmol g-1 FW)
(k)
Gly (nmol g-1 FW)
(l)
Total Amino Acids (nmol g-1 FW)
Rosette
Bud
Silique
Flower

Supplementary Figure 3. Cellular contents of amino acids in different organs of 6 wk-old WT and 2x*35S*::m*ODC*-1-7 transgenic plants. N= 3; each replicate consists of tissue from 2-3 individual plants. *= P≤ 0.05 for significant difference between WT and transgenic plants.

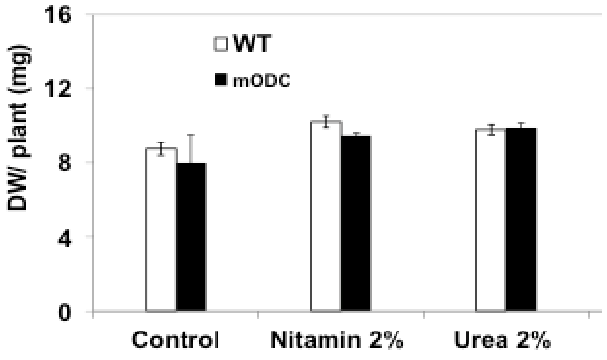

Supplementary Figure 4. Effect of foliar nitrogen treatments on the dry weight (DW) of mature (6 wk-old) WT and 2x*35S*::m*ODC Arabidopsis thaliana* plants. N=3 ± SE; *P* ≤0.05.

RNA Isolation, cDNA Synthesis and qPCR (Experiment 1; Data for Experiment 2 are presented in the main text)

Previously frozen plant samples (~200 mg FW in liquid nitrogen and stored at -80°C) were used for RNA extraction using ZR Plant RNA MiniPrep™Kit (Zymo Research, Irvine, CA) following the manufacturer's instructions. Samples were ground in a frozen (in liquid nitrogen) mortar with an autoclaved pestle (also frozen in liquid nitrogen), and mixed with 800 µl of Lysis buffer. Following thawing and centrifugation for 1 min at 12,000 xg, 600 µL of the supernatant was used for RNA purification using Zymo-Spin™IIIC column. After recommended washes, the column was treated with RNase-Free DNase (Zymo Research, Irvine, CA) at 37°C for 15 min. RNA was eluted in 25 µL of DNase/RNase-free water by centrifugation, quantified using NanoDrop 2000C (Thermo Scientific, Wilmington, DE), and either stored at -80°C or used immediately for cDNA preparation as follows.

For first strand cDNA synthesis, High-Capacity cDNA Reverse Transcription Kit (Applied Biosystems, Grand Island, NY) was used following the manufacturer's protocol. The final reaction volume of 20 µL contained 10 µL of 2X RT Master Mix and 10 µL of RNA sample (up to 2 µg of total RNA). The reaction was set up in an Eppendorf Mastercycler® thermal cycler (Eppendorf NA, Hauppauge, NY) with the following conditions: 10 min at 25°C, 120 min at 37°C and 5 min at 95°C. The cDNA was stored at -20°C for use in q-PCR analysis. All gene expression analyses reported here were done from the same RNA preparation but two sets of cDNA preparations.

Gene expression was quantified using Fast SYBR® Green Master Mix (Applied Biosystems, Grand Island, NY) following the manufacturer's protocol. Briefly, 5.5 µL of Fast SYBR green, a final concentration of 50 nmol each of gene-specific forward and reverse primers (Suppl. Table 1), and 100 ng of cDNA were mixed in a final reaction volume of 10 µL. The reactions were run in sub-skirted natural 96-well reaction plates (MIDSCI, Valley Park, MO) on the Applied Biosystems 7500 Fast Real-Time PCR thermocycler with the following conditions: initial denaturation at 95°C for 20 sec, and 40 cycles of denaturation at 95°C for 3 sec and annealing for

30 sec at 60°C followed by a dissociation step to confirm the single specific amplicon. A standard curve was generated using 4x serial dilution from 5x dilution of an original cDNA sample mixture. The standard curve was used to determine Ct values for gene expression quantification. The relative quantification to an internal control gene: *AtTIP*41- At4g34270 (Czechowski et al., 2005; Han et al., 2013) was done using the (Pfaffl, 2001) method. The data represent the results of Experiment 1, which did not have technical replicates. A select group of genes were analyzed using more rigorous approach and two technical replicates per biological replicate; those data are presented in the main body of the publication. The results using the two different approaches were quite consistent in showing that overproduction of Put via induction of mODC in the transgenic plants did not affect the transcriptome associated with the Glu-Orn-Arg-Pro-DAO-PAO and GAD pathway.

Supplementary Figure 5. The results of qPCR for the effects of inducible increase in Put production on changes in the relative expression of various genes of sub-pathways (a) Glu to Orn; (b) Orn to Arg; (c) Glu to Pro; and (d) PA catabolism and GABA biosynthesis in *Arabidopsis thaliana* using control and m*ODC* induced seedlings collected at 24 after induction with estradiol. The qPCR assays were conducted with three biological replicates per treatment. Data are mean ± SE of three biological replicates. For names of enzymes refer to legend of Figure 1 and Supplemental Table 1. *denotes significant difference (*P* ≤0.05) between m*ODC* induced seedlings and the corresponding un-induced control plants at the same time of analysis. However, only ≥2 fold differences with their respective controls are indicated as significant for biologically important changes.

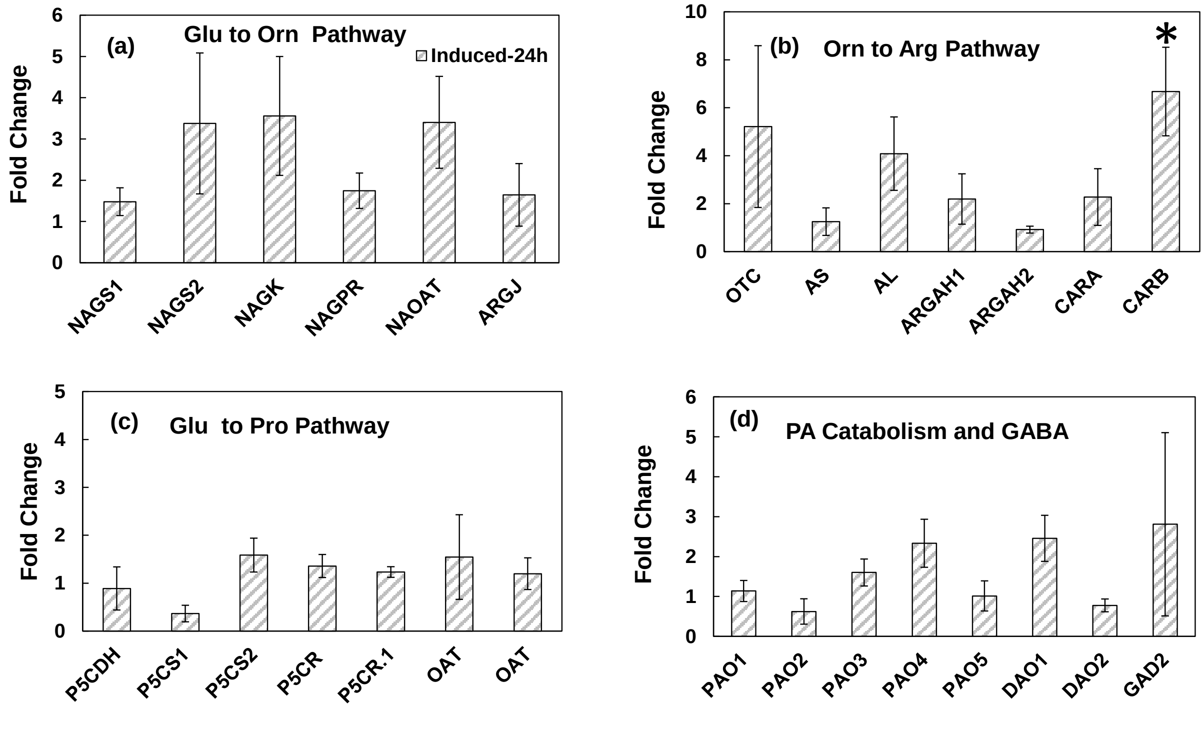

Supplementary Table 1 Primers used for expression analysis (QRT-PCR) of the genes involved in the biosynthesis of Orn, Arg, Pro, and GABA along with the polyamine catabolism genes in *Arabidopsis thaliana* (1-designed through NCBI using primer design tool; 2-Mattioli et al., 2009; 3-Takahashi et al. 2010; 4-Marina et al., 2013; 5-Page et al., 2007). **n.b.** *P5CR*.1 is a splice variant of *P5CR*.

Gene (Abbreviation)	Accession #	Forward Primers (5'-3')	Reverse Primers (5'-3')	Product size (bp)	Primer source
N-acetylglutamate synthase 1 (*NAGS*1; EC 2.3.1.1)	NM_127856.3	GCGTGGAGCTATGGTGGTAT	ATTCTCACCAGTTTCCGCGT	202	1
N-acetylglutamate synthase 2 (*NAGS*2; EC 2.3.1.1)	NM_119930	AGGCGATAAATTGCTCGGTGA	CGTCTCACAAACCAATCCGC	185	1
N-acetylglutamate kinase (*NAGK*; EC 2.7.2.8)	NM_115616	TCCCAGCTGAGTTTCGTGAC	GAACAGGTCGTGCTGTGAGA	177	1
N-acetylglutamate-5-phosphate reductase (*NAGPR*; EC 1.2.1.38)	NM_127552.7	GTCGAACTTCTCCGACCACT	AGCCTCTTGGCTCGCTTATC	160	1
*N*2- acetylornithine amino-transferase (*NAOAT*; EC 2.6.1.11)	NM_106708	TGTGCATCTGGAATCGCTGT	CTGCAAAAGAACTCGCCACC	160	1
*N*2-Acetylornithine:glu acetyl-transferase/bifunctional protein ArgJ (*NAOGAcT;* EC 2.3.1.35 and 2.3.1.1)	NM_129305.3	TCCTCATTTCTTCGCGCCTAA	CTCCACCAGCTATCTGTTTCCA	155	1
Ornithine transcarbamoylase (*OTC*; EC 2.1.3.3)	NM_106187	CGTATGGGCCAGTATGGGTC	TTCTCCGCCTGTGGAAAGAC	201	1
Argininosuccinate synthase (*AS*; EC 6.3.4.5)	NM_001125575	AATCTGGACTTCCAGTGGCG	CGGATCAAACCATCTTCCTCG	158	1
Argininosuccinate lyase (*AL*; EC 4.3.2.1)	NM_121130	GTAGAGCAGCTCGAACGTGA	AACTCCAGCACGAAGTCTCG	197	1
Arginase 1 (*ARGAH1*; EC 3.5.3.1)	NM_116959	GGCCGAAACAGAGATTTCGC	TCAATCACGCGATTTTGCCC	197	1
Arginase 2 (*ARGAH2*; EC 3.5.3.1)	NM_001036522.2	AAGAAGGACGGGAACAAGGC	CCCGAACTTTTCTTTCAAACCG	173	1
Carbamoyl phosphate synthetase (CPS:CARA; EC 6.3.5.5)	NM_113690	TCCTGGAACCCGTATTGCTG	TGTCTTCGTGCATCTCCAGT	226	1
Carbamoyl phosphate synthetase (CPS:CARB; EC 6.3.5.5)	NM_102730	AGCCATTGGACACCCTCTTG	TCTCTGGCCCGAGTATCACA	167	1

Δ[1]-pyrroline-5-carboxylate dehydrogenase (*P5CDH*; EC 1.5.1.12)	NM_125647.3	AGAGCTAAGTCTCTCTGTGAC	CTTTCCCTGCACAAAGCTC	150	1
Δ[1]-pyrroline-5- carboxylate synthetase 1 (*P5CS*1; EC 2.7.2.11/1.2.1.41)	NM_129539	CGACGGAGACAATGGAATTGT	GCTTGGATGGGAATGTCCTG	50	2
Δ[1]-pyrroline-5- carboxylate synthetase 2 (*P5CS*2; EC 2.7.2.11/1.2.1.41)	NM_115419.4	CACCCATAAGGATCTTCCTGTC TT	CCATTCTCAACAGCCTCTGTCC	50	2
Δ[1]-pyrroline-5-carboxylate reductase (*P5CR*; EC 1.5.1.2)	NM_121484.4	AGCAATTGAAGCTTTAGCCG	TCCAAGAACGGTCTGTGAAG	88	1
Δ[1]-pyrroline-5-carboxylate reductase (*P5CR.1*; EC 1.5.1.2)	NM_001085115.1	CACAGACCGTCAGTTCTTGGA	CCGCCAGGTGAGGTAACAT	92	1
Proline dehydrogenase (*ProDH*; EC 1.5.99.8)	NM_113981	TCTTCTCCGACGCGCTTATG	TTCCATCCTCATGAGTTGACGG	76	1
Ornithine δ-aminotransferase (*OAT*; EC 2.6.1.13)	NM_123987	GGCTAGATCCGGGAAGATGC	CCACCAAATGTGCTTCCGTG	168	1
Polyamine oxidase 1 (*PAO*1; EC 1.5.3.11)	NM_121373	TGCCGGTGTCGGTGGTA	GGTTAAAACGCGAGGCAAGT	59	3
Polyamine oxidase 2 (*PAO*2; EC 1.5.3.11)	NM_129863	GATCCTTTGGCCCCCATT	TACGACGATCGGCGATATCTG	58	3
Polyamine oxidase 3 (*PAO*3; EC 1.5.3.11)	NM_115767	CCGTTCATTGTTGAATCATGGA	AATAGCTTTACGCAGCTGACGAT	62	3
Polyamine oxidase 4 (*PAO*4; EC 1.5.3.11)	NM_105256	GCGAACGAAGCCGTTATT	CAATGGCGAGGATTCAAAAAC	79	3
Polyamine oxidase 5 (*PAO*5; EC 1.5.3.11)	NM_119117	CCGGGAGGATTCCGAGTT	ATGGTCGCGGTTCTTCTCAT	63	3
Diamine oxidase 1 (*DAO*1/ *ATAO1*; EC 1.4.3.22)	At4g14940 (TAIR locus ID)	CTCGCGTCAAGTTCCAAGAC	TCAGGTGTCGTTAGTGGGTCAAG	100	4
Diamine oxidase 2 (*DAO*2/*ATAO2*; EC 1.4.3.22)	At1g31690 (TAIR locus ID)	GAAGGACCTGCCTCTGTACCT	ATTAAGACAATCATCACTTGAGG TTCC	100	4
Glutamate decarboxylase 2 (*GAD*2; EC 4.1.1.15)	NM_001124084.1	AAACCGCAACGAATGATGAA	TCTCATACTTGGGAAGTGTAGT	78	1
Mouse ornithine decarboxylase (m*ODC*; EC 4.1.1.17)	NM_013614.2	TGGCCTTGATCGGATCGT	TCCAATCACCCACATGCATT	59	5
TIP41-like family protein (*TIP*41; internal control gene)	NM_119592	CGAGGTTTACGCATCCATGA	TCGACAGCGAGAGAAGTGAGAA	71	5

Supplementary Table 2. Detailed data on QPCR analyses with technical replicates for results shown in Figure 8. For more details see legend to Figure 8 and Materials and Methods.

**Induced 24 h**															
Biol. Reps	Techn. Reps	OTC	GAD2	ARGAH1	ARGJ	CARB	PAO2	ProDH	PAO3	PAO4	CARA	DAO1	DAO2	NAGK	NAOAT
B1	Q1	1.85	2.97	4.14	3.61	1.30	0.15	0.10	1.00	0.04	0.03	1.00	0.48	32.00	40.22
	Q2	NA	3.10	3.73	5.70	1.27	0.10	0.09	0.26	1.00	0.02	6.32	0.70	32.00	30.48
B2	Q1	NA	1.72	1.76	2.62	1.46	1.00	0.53	1.00	0.83	1.33	2.89	0.58	1.61	1.81
	Q2	1.16	1.64	1.81	2.87	1.40	0.79	0.54	1.00	0.79	1.30	3.12	0.57	1.69	1.84
B3	Q1	1.38	1.67	1.26	1.43	1.46	3.41	2.73	3.68	2.28	3.27	1.18	1.40	1.03	1.01
	Q2	1.50	2.08	1.11	1.29	1.19	3.32	2.30	3.63	2.35	3.63	1.11	1.30	1.01	1.01
Mean (N=3)		1.48	2.20	2.30	2.92	1.35	1.46	1.05	1.76	1.22	1.60	2.60	0.84	11.56	12.73
SE		0.20	0.42	0.84	0.95	0.04	0.98	0.75	0.95	0.56	1.00	0.75	0.26	10.22	11.32
**Induced 48 h**		OTC	GAD2	ARGAH1	ARGJ	CARB	PAO2	ProDH	PAO3	PAO4	CARA	DAO1	DAO2	NAGK	NAOAT
B1	Q1	1.01	0.89	0.71	0.82	1.34	0.90	1.13	0.93	0.61	1.07	2.46	2.08	0.88	0.91
	Q2	1.23	1.00	0.92	0.76	0.14	0.86	1.46	0.87	0.69	1.00	2.50	1.00	0.89	1.04
B2	Q1	1.15	1.06	2.06	1.06	0.06	1.27	0.61	1.30	1.01	0.38	1.22	0.90	1.66	1.42
	Q2	1.15	0.88	2.45	1.16	NA	1.61	0.42	1.45	1.19	1.00	0.78	1.00	1.66	1.51
B3	Q1	0.74	1.07	1.10	1.26	0.65	0.70	0.56	1.44	1.22	0.83	1.45	0.28	1.03	1.00
	Q2	1.07	1.22	1.04	1.23	NA	0.70	0.67	1.20	1.13	1.00	1.31	1.00	1.02	0.91
Mean (N=3)		1.03	1.02	1.09	0.99	0.72	0.89	0.93	1.11	0.88	0.89	1.90	1.22	1.06	1.03
SE		0.08	0.07	0.23	0.13	0.62	0.10	0.16	0.12	0.16	0.11	0.31	0.44	0.11	0.10
